# Systems-wide analysis of manganese deficiency-induced changes in gene activity of *Arabidopsis* roots

**DOI:** 10.1038/srep35846

**Published:** 2016-11-02

**Authors:** Jorge Rodríguez-Celma, Yi-Hsiu Tsai, Tuan-Nan Wen, Yu-Ching Wu, Catherine Curie, Wolfgang Schmidt

**Affiliations:** 1 Institute of Plant and Microbial Biology, Academia Sinica, 128 Academia Road, Taipei, Taiwan; 2 Biochimie et Physiologie Moléculaire des Plantes, Centre National de la Recherche Scientifique, Institut National pour la Recherche Agronomique, Laboratoire de Biochimie et Physiologie Moléculaire des Plantes, INRA/SupAgro, Université Montpellier 2, Montpellier, France; 3 Graduate Institute of Biotechnology, National Chung Hsing University, Taichung, Taiwan; 4 Genome and Systems Biology Degree Program, College of Life Science, National Taiwan University, Taipei, Taiwan

## Abstract

Manganese (Mn) is pivotal for plant growth and development, but little information is available regarding the strategies that evolved to improve Mn acquisition and cellular homeostasis of Mn. Using an integrated RNA-based transcriptomic and high-throughput shotgun proteomics approach, we generated a comprehensive inventory of transcripts and proteins that showed altered abundance in response to Mn deficiency in roots of the model plant *Arabidopsis*. A suite of 22,385 transcripts was consistently detected in three RNA-seq runs; LC-MS/MS-based iTRAQ proteomics allowed the unambiguous determination of 11,606 proteins. While high concordance between mRNA and protein expression (R = 0.87) was observed for transcript/protein pairs in which both gene products accumulated differentially upon Mn deficiency, only approximately 10% of the total alterations in the abundance of proteins could be attributed to transcription, indicating a large impact of protein-level regulation. Differentially expressed genes spanned a wide range of biological functions, including the maturation, translation, and transport of mRNAs, as well as primary and secondary metabolic processes. Metabolic analysis by UPLC-qTOF-MS revealed that the steady-state levels of several major glucosinolates were significantly altered upon Mn deficiency in both roots and leaves, possibly as a compensation for increased pathogen susceptibility under conditions of Mn deficiency.

Manganese (Mn) is an essential mineral nutrient for all organisms, playing important roles in the detoxification of reactive oxygen species and, often as a co-factor of important enzymes, in a multitude of biosynthetic pathways^1^. In plants, Mn is also critical for the function of photosystem II as a component of oxygen-evolving complex (OEC), which catalyzes the oxidation of water to protons and molecular oxygen. Manganese deficiency reduces plant growth, increases susceptibility to infection^2^,^3^ and induces PSII photoinhibition by destabilizing the cubane–like Mn cluster in the OEC, thereby compromising photosynthetic activity^4^,^5^. Manganese deficiency is a worldwide problem that is particularly severe in Australia, the northern parts of the United States and Canada, and in the northern parts of Europe^6^. In particular sandy soils, soils rich in organic matter and soils with high pH restrict the bioavailability of Mn.

The uptake of Mn across various organisms is mediated by Nramps (natural resistance-associated macrophage proteins), a group of integral membrane transporters that are highly conserved among bacteria, fungi, and animals. In *Arabidopsis*, NRAMP1 was shown to be critical for Mn uptake; loss of NRAMP1 function led to severe growth reduction and decreased Mn concentrations^7^. In support of this finding, screening of *Arabidopsis* accessions for reduced photosynthesis under chilling temperature identified a mutation in a conserved histidine in the NRAMP1 allele of the Hog accession as the cause for the observed phenotype^8^. In rice, OsNRAMP5 mediates the trans-plasma membrane transport of Mn (and Fe) from the soil^9^. In *Arabidopsis*, two other Nramp proteins, NRAMP3 and NRAMP4, are critical in remobilizing vacuolar Mn (and Fe) in leaves prior to the import into chloroplasts of mesophyll cells, and are required for functional PSII^10^. In addition to Nramps, several ZIP (*Z*RT/*I*RT-like *P*roteins) transporters were shown to transport Mn in yeast complementation assays^11^. The ZIP protein AtIRT1 mediates trans-plasma membrane transport of several transition metals such as Cd, Ni, Co and Mn^12^ and the expression level of its ortholog in barley correlates with root Mn uptake efficiency^13^. ZIP1 and ZIP2 have been associated with Mn translocation from the root to the shoot, but both transporters are unlikely to be involved in the uptake of Mn from the soil^11^. An overview of the Mn-transporting enzymes is provided in a recent review by Socha and Guerinot^14^.

In contrast to other soil-immobile nutrients such as phosphate and Fe for which the mechanisms that re-calibrate cellular homeostasis are relatively well explored, only fragmentary information is available regarding the responses of plants to low Mn supply. Such knowledge is of critical importance for the development of Mn-efficient germplasm with improved Mn acquisition and/or increased resource utilization efficiency. Transcriptome profiling has allowed insights into Mn deficiency-induced changes of gene activity^15^; however, biological processes are carried out by proteins and it is not clear how much the changes observed at the transcript level impact the proteomic readout. In fact, several studies observed only a moderate level of concordance between transcriptomics and proteomics (reviewed by Vogel and Marcotte^16^), indicating the necessity of integrative studies that cover disparate omics levels.

In the present study, we undertook a comprehensive quantitative profiling of gene activity in roots from plants grown on Mn-deplete media, aiming at dissecting and cataloging the responses of *Arabidopsis* roots to Mn deficiency. To acknowledge the generally observed moderate correlation of changes in transcript and protein abundance, we opted for an integrative transcriptomic and proteomic study. We used ultra high pressure liquid chromatography on a Q Exactive Orbitrap mass spectrometer to compile a near complete inventory of the *Arabidopsis* root proteome. To compare and integrate proteomic changes with alterations in the transcriptome, we investigated such changes by RNA-seq on an Illumina HiSeq 2000 platform. To validate changes observed in the activity of enzymes involved in glucosinolate metabolism at the transcript and protein level, we investigated changes in specific glucosinolates by UPLC-qTOF-MS. We identified several novel components of cellular Mn homeostasis and show that many putatively important changes in response to Mn deficiency are only evident at one of the omics levels under investigation.

## Methods

### Plant growth conditions

*Arabidopsis* (*Arabidopsis thaliana* (L.) Heynh) plants were grown in a growth chamber on solid medium as described by Estelle and Somerville^17^. Seeds of the accession Columbia (Col-0) were obtained from the Arabidopsis Biological Resource Center (Ohio State University). Seeds were surface-sterilized by immersing them in 5% (v/v) NaOCl for 5 min and 70% ethanol for 7 min, followed by four rinses in sterile water. Seeds were placed onto Petri dishes and kept for 1 d at 4 °C in the dark, before the plates were transferred to a growth chamber and grown at 21 °C under continuous illumination (50 μmol m^−2 ^s^−1^; Phillips TL lamps). The medium was composed of (mM): KNO_3_ (5), MgSO_4_ (2), Ca(NO_3_)_2_ (2), KH_2_PO_4_ (2.5) and (μM): H_3_BO_3_ (70), ZnSO_4_ (1), CuSO_4_ (0.5), Na_2_MoO_4_ (0.2), CoCl_2_ (0.01), FeEDTA (40), 1% (w/v) MES, supplemented with sucrose (43.8 mM) and solidified with 0.8% plant cell culture tested agar (Sigma A7921). The pH was adjusted to 5.5. Plants were grown for 14 d in media supplemented with 14 μM MnCl_2_ (+Mn) or without Mn (−Mn).

### RNA sequencing

Three independently grown batches of plants grown under Mn-sufficient and Mn-deficient conditions were used for analysis. For each sample, roots from 10 Mn-sufficient and 20 Mn-deficient plants were pooled and total RNA was extracted from roots or leaves using the RNeasy Plant Mini Kit (Qiagen), following manufacturer instructions. Nucleic acid quantity was analyzed with a NanoDrop ND-1000 UV-Vis Spectrophotometer (NanoDrop Technologies, Wilmington, USA). For RNA-seq, equal amounts of total RNA were collected and cDNA libraries for sequencing were prepared from total RNA following the manufacturer’s protocol (Illumina). The cDNA libraries were subsequently enriched by PCR amplification. The resulting cDNA libraries were subjected to sequencing on a single lane of an Illumina HiSeq 2000 machine. RNA-seq and data collection was done following the protocol of Mortazavi *et al*.^18^. The length of the cDNA library was maintained from 250 to 300 bp with a 5′-adapter of 20 bp and a 3′-adapter of 33 bp at both ends. Eventually, the fragment length range of the cDNA was 200 to 250 bp. To quantify gene expression levels, 75-mers sequences were aligned to the genomic sequence annotated in TAIR10 using the BLAT program^19^ and RPKM (Reads Per Kbp per Million reads) values were computed using the RACKJ (Read Analysis & Comparison Kit in Java, http://rackj.sourceforge.net/) software.

### Bioinformatics

Gene clustering was performed using the MACCU software package (http://maccu.sourceforge.net/) to build co-expression clusters based on pairwise co-expression relationships of genes with Pearson coefficients greater than or equal to 0.75. In order to capture the co-expression relationships specifically in roots, Pearson coefficients were computed based on robust multi-array averaged array data derived from publically available root-specific experiments downloaded from NASCArrays (http://affymetrix.arabidopsis.info/). Visualization of the networks was performed with the Cytoscape software version 3 (http://www.cytoscape.org/).

### qRT-PCR

Shoot and root samples (pooled from 10–20 seedlings from three independently grown batches of plants) were collected from 14-day-old seedlings and frozen immediately in liquid nitrogen. Total RNA was isolated using RNeasy Mini Kit (Qiagen) and treated with DNase using TURBO DNA-*free* Kit (Ambion) as indicated by the manufacturer. cDNA was synthesized using DNA-free RNA with Oligo-dT (20) primers and SuperScript III First-Strand Synthesis System for RT-PCR (Invitrogen). After incubation at 50 °C for 1 h and subsequently at 70 °C for 15 min, 1 μL of RNase H was added and incubated for 20 min at 37 °C. The cDNA was used as a PCR template in a 20 μL reaction system using the SYBR Green PCR Master Mix (Applied Biosystems) with programs recommended by the manufacturer in an AB QuantStudio Real Time PCR System (Applied Biosystems). Three independent replicates were performed for each sample. The ∆∆C_T_ method was used to determine the relative amount of gene expression^20^, with the expression of elongation factor 1 (EF1) used as an internal control. The following primers were used: *EF1A*, 5′-AACTTTGATGGCGTTTGAGC-3′ and 5′-TCCGGAAACTGCATAATTGA-3′; *BGLU23*, 5′-CATTGGTAGCAAGCCTTTGA-3′ and 5′-ATGATCAGCGGTACCAACAG-3′; *TTG1*, 5′-AATGGGATTGATCCGATGTC-3′ and 5′-CACTTCACATCTGCACCTCA-3′.

### ICP-OES

Fifteen to 20 shoot samples were collected from 14-day-old seedlings (yielding 0.03 to 0.05 g dry weight), washed with 10 mM CaCl_2_ and H_2_O, dried at 60 °C for 3 d, and weighed before microwave-digestion. Samples (50 mg) were digested with 5 mL of 65% HNO_3_ and 2 mL H_2_O_2_ in a MarsXpress microwave digestion system (CEM, Matthews, NC, USA). The volume of digested samples was adjusted to 10 mL with H_2_O and filtered using a 0.45 μm membrane filter. Tomato leaves (SRM-1573a) from the National Institute of Standards and Technology (Gaithersburg, MD, USA) were used as a standard. The concentration of Mn in digested samples was analyzed by inductively coupled plasma-optical emission spectrometry (ICP-OES; OPTIMA 5300, Perkin-Elmer, Wellesley, MA, USA). The Mn concentration for each sample was determined by triplicate measurements.

### Protein extraction

Three independently grown batches of plants grown under Mn-sufficient and Mn-deficient conditions were used for analysis. Roots from 100 Mn-sufficient plants and 200 Mn-deficient plants (14-day-old) were pooled, ground in liquid nitrogen and suspended in 10 × volume of precooled acetone (−20 °C) containing 10% (v/v) TCA and 0.07% (v/v) 2-mercaptoethanol. Proteins were then precipitated for 2 h at −20 °C after thorough mixing. Precipitated proteins were collected by centrifuging at 35,000 *g* (JA-20 108 rotor; Beckman J2-HS) at 4 °C for 30 min. The supernatant was carefully removed, and the protein pellets were washed twice with cold acetone containing 0.07% (v/v) 2-mercaptoethanol and 1 mM phenylmethanesulfonyl fluoride (PMSF) and a third time with cold acetone. Protein pellets were dried and stored at −80 °C or immediately dissolved using protein extraction buffer composed of 8 M urea, 50 mM triethylammonium bicarbonate, pH 8.5, for 1 h at 6 °C under constant shaking. Protein extracts were centrifuged at 19,000 *g* for 20 min at 10 °C. The supernatant was then collected, and the protein concentration was determined using a protein assay kit (Pierce).

### In-solution trypsin digestion and iTRAQ labeling

Total protein (100 μg) was digested and iTRAQ labeled as described elsewhere^21^. Subsequently, iodoacetamide was added to a final concentration of 50 mM, and the mixture was incubated for 30 min at room temperature in the dark. Then, dithiothreitol (30 mM) was added to the mixture to consume any free iodoacetamide by incubating the mixture for 1 h at room temperature in the dark. Proteins were then diluted by 50 mM Tris, pH 8.5, to reduce the urea concentration to 4 M, and digested with 0.5 μg of Lys-C (Wako) for 4 h at room temperature. After digestion, the solution was further diluted with 50 mM Tris, pH 8.0, to reduce the urea concentration to less than 1 M. The Lys-C digested protein solution was further digested with 2 μg of modified trypsin (Promega) at room temperature overnight. The resulting peptide solution was acidified with 10% trifluoroacetic acid and desalted on a C18 solid-phase extraction cartridge.

Desalted peptides were then labeled with iTRAQ reagents (Applied Biosystems) according to the manufacturer’s instructions. Control samples (proteins extracted from roots of control plants) were labeled with reagent 114; samples from Mn-deficient roots were labeled with reagent 116. Three independent biological experiments with two technical repeats each were performed. The reaction was allowed to proceed for 1 h at room temperature. Subsequently, treated and control peptides were combined and further fractionated offline using high-resolution strong cation-exchange chromatography (PolySulfoethyl A, 4.6 × 100 mm, 5 μm, 200-Å bead). In total, 60 fractions were collected and combined into 24 final fractions. Each final fraction was desalted on a C18 solid-phase extraction cartridge and lyophilized in a centrifugal speed vacuum concentrator. Samples were stored at −80 °C.

### LC-MS/MS analysis

Liquid chromatography was performed on a Dionex UltiMate 3000 RSLCnano System coupled to a Q Exactive hybrid quadrupole-Orbitrap mass spectrometer (Thermo Scientific) equipped with a Nanospray Flex Ion Source. Peptide mixtures were loaded onto a 75 μm × 250 mm Acclaim PepMap RSLC column (Thermo Scientific) and separated using a segmented gradient in 120 min from 3 to 30% solvent B (100% acetonitrile with 0.1% formic acid) at a flow rate of 300 nL/min. Solvent A was 0.1% formic acid in water. The samples were maintained at 8 °C in the autosampler. The Orbitrap was operated in the positive ion mode with the following acquisition cycle: a full scan (*m/z* 350~1600) recorded in the Orbitrap analyzer at resolution R 70,000 was followed by MS/MS of the 10 most intense peptide ions with HCD of the same precursor ion. HCD collision energy was set to 30% NCE. HCD-generated ions were detected in the Orbitrap at resolution 17,500.

### Database search

Two search algorithms, Mascot (version 2.4, Matrix Science) and SEQUEST, which is integrated in Proteome Discoverer software (version 1.4, Thermo Scientific), were used to simultaneously identify and quantify proteins. Searches were made against the Arabidopsis protein database (TAIR10 20110103, 27416 sequences; ftp://ftp.arabidopsis.org/home/tair/Sequences/blast_datasets/TAIR10_blastsets/ TAIR10 pep 20110103 representative gene model) concatenated with a decoy database containing the reversed sequences of the original database. The protein sequences in the database were searched with trypsin digestion at both ends and two missed cleavages allowed, fixed modifications of carbamidomethylation at Cys, iTRAQ 4plex at N-terminus and Lys, variable modifications of oxidation at Met and iTRAQ 4plex at Tyr; peptide tolerance was set to 10 ppm, and MS/MS tolerance was set to 0.05 Da. iTRAQ 4plex was chosen for quantification during the search simultaneously. The search results were passed through additional filters, peptide confidence more than 95% (*P* < 0.05), before exporting the data. For protein quantitation, only unique peptides were used to quantify proteins. These filters resulted in a false discovery rate of less than 5% after decoy database searches were performed. For each of the three biological repeats, spectra were combined into one file and searched. Annotated spectra of proteins and peptides identified in roots and leaves (available at ProteomeXchange Consortium; http://www.ebi.ac.uk/pride; dataset identifier PXD003309).

### Statistical analysis

Proteins with significant changes in abundance upon Mn deficiency were selected using a method described by Cox and Mann^22^. In brief, the mean and SD from the log2 ratios of the defined 11,606 quantified proteins was calculated. Next, 95% confidence (Z score = 1.96) was used to select those proteins whose ratio was significantly different from the main distribution (mean ratio ± 1.96 × SD), protein ratios outside this range were defined as being significantly different at *P* < 0.05. The cutoff value for down-regulated proteins was 0.75-fold and for up-regulated proteins 1.39-fold.

### Glucosinolate analysis

Glucosinolates were analyzed using a protocol described by Glauser *et al*.^23^. Samples were collected from roots and leaves of 14-day-old seedlings and frozen immediately in liquid nitrogen. Samples (200 mg) were shattered into powder with TissueLyser II (QIAGEN) and 1 mL of freshly prepared ice-cold methanol:water (70:30, v/v) with sinalbin (Applichem) as internal standard. After homogenization for 30 s with TissueLyser II, samples were incubated for 15 min at 80 °C in a block heater (BL3002, Basic Life), cooled down to room temperature and centrifuged at 3,500 *g* for 10 min. The supernatant was transferred to appropriate vials for UPLC-qTOF analysis. UPLC-qTOF-MS analysis was performed on an ACQUITY UPLC (Waters, Manchester, UK) and a SYNAPT G1 High Definition Mass Spectrometry (Synapt G1 HDMS) System (Waters) with an electrospray ionization interface, ion-mobility and time-of-flight system. Samples were separated on an ACQUITY CSH C18 column (2.1 × 100, 1.7 μm; Waters). The mobile phase consisted of 0.05% formic acid in 2% acetonitrile (solution A) and 0.05% formic acid in 100% acetonitrile (solution B). The elution gradient was as follows: starting with 1% B for 2 min, 1–45% B for 6 min, 45–99% B for 1 min, holding at 99% B for 1 min, 99–1% B in 0.1 min and then holding at 1% B for 0.9 min. The flow rate was 0.4 mL/min, and the column temperature was set to 25 °C. The injected volume was 2 μL. Spectra were collected in the negative ionization and V mode setting. The electrospray capillary voltage was set to 2.5 kV and the cone voltage was set to 40 V. The source and desolvation temperatures were 80 °C and 250 °C. The desolvation and cone gas flow rates were 800 L/h and 50 L/h. A lock mass calibration of sulfadimethoxin at a concentration of 0.05 mg/L in water:methanol (50:50, v/v) was introduced by HPLC pump (LC-10ATVP, Shimadzu, Japan) and split the 60 μL/min to lock spray interface. The MS range acquired was 50–1200 *m/z* with 0.2 sec/scan in the centroid mode. For data analysis, the acquired mass data were imported to Markerlynx (Waters) within the Masslynx software (version 4.1) for peak detection and alignment. The retention time and *m/z* data for each peak were determined by the software. All data were normalized to the summed total ion intensity per chromatogram.

## Results

### Mn deficiency-induced changes in gene activity

**Figure 1:**
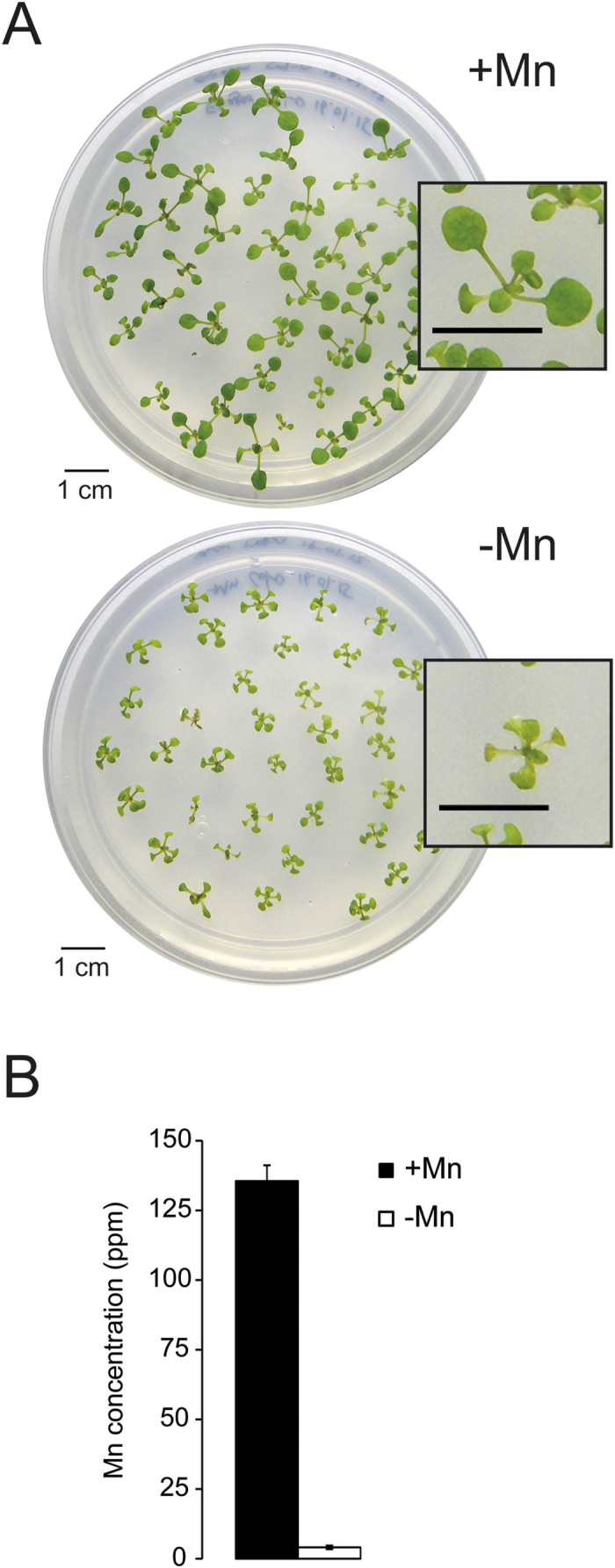
Phenotype of Mn-deficient plants. (**A**) *Arabidopsis* plants after 12 days of growth on Mn-replete (+Mn) and Mn-deplete (−Mn) media. (**B**) Mn concentrations in shoots of +Mn and −Mn plants. Error bars show standard deviations from the mean of three experimental runs.

**Figure 2:**
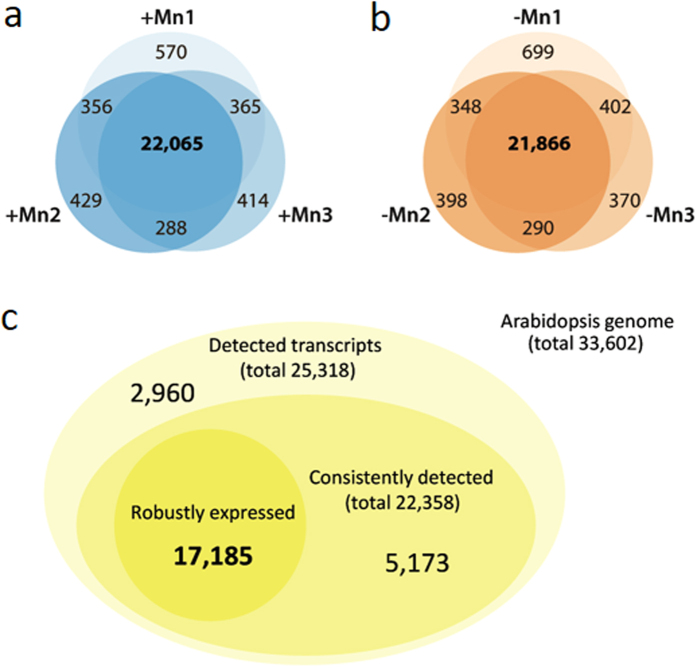
Detection of mRNAs by RNA-seq. (**a**,**b**) Detected transcripts in three independent experiments with Mn-sufficient (+Mn) or Mn-deficient (−Mn) *Arabidopsis* roots. (**c**) Total, consistently and robustly detected transcripts in roots of both growth types.

Plants grown on Mn-free media showed reduced growth, pronounced leaf chlorosis and significantly reduced Mn levels (Fig. 1). It is important to note that manganese-deplete medium was prepared with plant cell culture tested agar to avoid Mn contaminations introduced by other gelling agents. To identify Mn-responsive genes in *Arabidopsis* roots, we first surveyed transcriptional profiles of Mn-sufficient and Mn-deficient plants by RNA-seq. Experiments were performed in triplicates with more than 12 million unique reads per library (37.7 and 37.4 million unique reads for +Mn and −Mn plant, respectively). With this approach, we identified a total of 25,318 transcripts of which 22,385 were detected in all three replicates, a subset that we refer to as consistently detected transcripts (22,065 in Mn-sufficient roots and 21,866 in Mn-deficient roots; Fig. 2). To exclude noisy genes that are likely of minor importance for the processes under study, we defined biologically relevant expression based on data distribution. Only transcripts with a RPKM value larger than the square root of the median of the distribution of the RPKM values of all detected genes (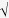
10.95 = 3.31) were considered for further analysis. Applying this filter yielded a subset of 17,185 genes that were defined as robustly expressed. A subset of 5,173 genes was consistently detected but at low expression levels and was not further considered here (Fig. 2c).

To gain a comprehensive picture of the Mn deficiency responses, we complemented the catalog of expressed transcripts with a proteomic survey using the iTRAQ methodology. iTRAQ-labeled peptides were analyzed by liquid chromatography in combination with tandem mass spectroscopy on a Q Exactive hybrid quadrupole-Orbitrap mass spectrometer, using the Mascot and SEQUEST search algorithms to identify the proteins. With this approach, we identified a total of 22,843 proteins, of which 11,606 could be consistently and unambiguously determined in at least two out of three parallel runs. Read numbers (RNA-seq) and fold-changes (iTRAQ) for all detected transcripts and proteins are given in Supplemental Table S1.

### Detection of differentially expressed transcripts and proteins

**Figure 3:**
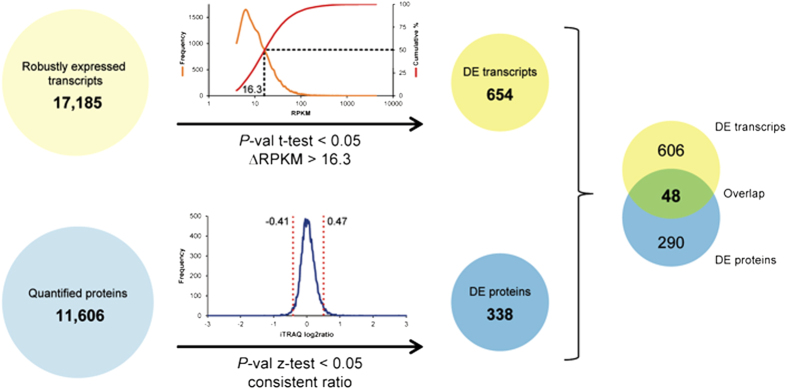
Detection of differentially expressed genes and proteins. Genes that were statistically relevant (*P* < 0.05) and with a ∆RPKM between Mn-deficient and Mn-sufficient plants greater than the median of the RPKM values of all robustly expressed genes were referred to as differentially expressed (DE transcripts). For proteins, a change in the abundance in the same direction in all three biological replicates and a *P*-value < 0.05 (Z-test) was used as criteria to define differential expression (DE proteins).

To prioritize genes with potentially high significance for the acclimation to Mn deficiency, we defined transcripts with a ∆RPKM between the two growth conditions greater than the median of the distribution of the RPKM values of all robustly expressed genes (16.3) and a *P*-value < 0.05 (Student’s t-test) as differentially expressed (DE transcripts). Applying these filters yielded 654 DE transcripts (Fig. 3). Since the quantification of protein abundance determined by the iTRAQ technology is defined by the ratio between Mn-deficient and Mn-sufficient plants and not by absolute values, we applied a Z-test (*P* < 0.05) to define differentially expressed proteins. Only proteins with abundance changed in the same direction in all three biological replicates (either increased or decreased abundance in response to Mn deficiency) were considered. These criteria yielded a subset of 338 DE proteins with significantly altered abundance upon Mn deficiency out of a total of 11,606 identified proteins that could be quantified (Fig. 3).

### Concordance of mRNA and protein abundance

**Figure 4:**
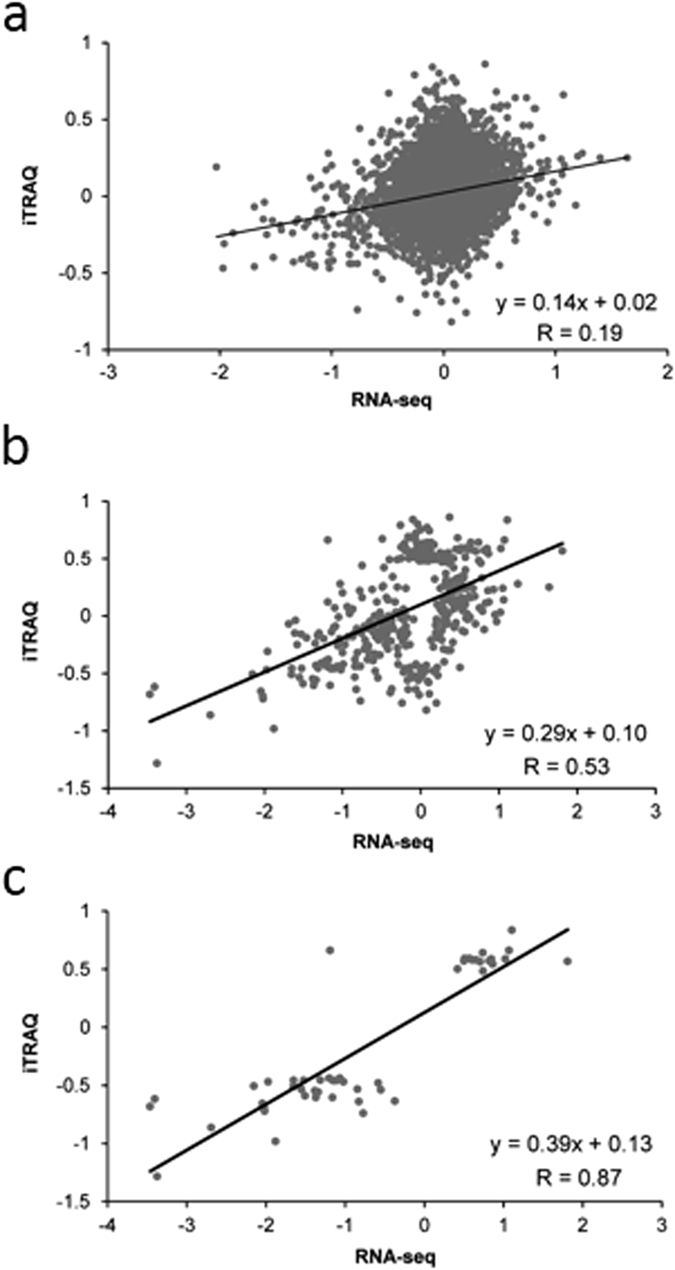
Concordance of mRNA and protein expression. (**a**) Pearson correlation of all detected mRNAs and proteins. (**b**) Concordance calculated with DE transcripts/proteins independent of the regulation of the cognate partner. (**c**) Concordance of genes/protein pairs for which both partners were differentially expressed.

A comparison of DE transcripts and DE proteins revealed a relatively small overlap of 48 genes that were differentially expressed between Mn-sufficient and Mn-deficient plants at both levels (Fig. 3; Table 1; Supplemental Table S2). Discordance of mRNA and protein expression may result from differences in mRNA/protein synthesis and stability or may be caused by other post-transcriptional processes. Notably, only 14% of the differentially expressed proteins were accompanied by a cognate transcript that was changed in the same direction, indicative of a large contribution of post-transcriptional regulation of gene activity (Fig. 3). Due to the unbiased nature of the RNA-seq technology it is unlikely that a larger fraction of differentially expressed mRNAs was not detected. As anticipated from this small overlap, transcript and protein abundance was only weakly related to each other when all detected mRNAs and proteins were considered (R = 0.19; Fig. 4a). When DE transcripts/DE proteins were used as input without considering whether the cognate partner was differentially expressed, a moderate correlation (R = 0.53) was observed (Fig. 4b). The correlation coefficient was significantly increased when only the 48 genes that constitute the overlap of the DE mRNAs/proteins were analyzed, *i.e.* in the population in which both partners were defined as differentially expressed (R = 0.87; Fig. 4c). Thus, it appears that the two data sets are highly discordant except for those genes that were differentially expressed at both the protein and at the transcript level. If this is the case, most (~90%) of the changes in protein abundance can be explained by changes in gene transcription. The data also allow the conclusion that most genes do not follow the anticipated pattern of parallel change of mRNA and protein abundance. In the majority of cases, the change in either transcript or protein abundance was not associated with changes of the cognate partner.

### The ‘Manome’ is regulated at different layers

Genes that were differentially expressed between Mn-sufficient and Mn-deficient plants could be categorized into: 1) genes for which a simultaneous change in transcript and protein abundance in the same direction was observed; 2) post-transcriptionally regulated genes, *i.e.* genes that were only regulated at the protein level or transcript and protein were regulated in opposite directions, and 3) genes that were solely regulated at the transcriptional level without changes in the corresponding protein. Since changes in the activity of genes from the first group can be tracked at two different levels and thus have low gene expression noise, we defined these genes as being robustly Mn-regulated. From the 48 genes in this group (Supplemental Table S2), only few have been associated with Mn homeostasis. *NRAMP1*, the main route of entry for Mn from the soil^7^, was moderately up-regulated in roots of Mn-deficient plants both at the mRNA and protein level. Also CHLORIDE CHANNEL A (CLCA), mediating the transport of nitrate into the vacuole^24^, was up-regulated at both levels. The majority of robustly Mn-regulated genes was, however, down-regulated in response to Mn deficiency. Most of the genes with reduced transcript abundance are related to photosynthesis (*PSAK*, *PSAN*, *PSAE-1*, *PSAD-1*, *PSBQ-2*, *PSBO-2*, *CAB3*) or are involved in the response to hypoxia (*ADH1*, *PDC1*, *PCO1*, *HB1*, At4g27450). Also, expression of the metallothionein *MT2B* was reduced upon Mn deficiency.

A subset of 290 proteins was classified as chiefly or entirely post-transcriptionally regulated (Supplemental Table S2). Interestingly, in this group a more pronounced regulation of the protein abundance was observed, spanning a range from 0.5-fold down- to 6.8-fold up-regulation, while that of the robustly Mn-regulated genes was much narrower (0.41 to 1.78-fold). Several up-regulated genes in this group are related to translation (*EIF4A-III*, *EIF4A-2*, *ERF1-1*, *EF1B*, At2g45030), mRNA stability (*PUM7*), mRNA modification (At1g61470), or mRNA splicing (*SC35*, *LUC7*, *PRP38*, At2g16940, At2g29210). Splicing-related genes were regulated in a complex pattern; while the *Arabidopsis ORTHOLOG OF HUMAN SPLICING FACTOR* (*SC35*) was up-regulated upon Mn deficiency, the SC35-like splicing factors (SCLs) *SCL30* and *SCL30A* showed reduced abundance in roots of Mn-deficient plants, suggesting Mn deficiency-induced alterations in the composition of the splicing machinery. Several translation-associated proteins were increased in abundance when plants were grown under conditions of Mn-deficiency. Notably, none of the translation-associated gene was transcriptionally regulated by Mn deficiency. Similar to what has been observed for *MT2B*, the metallothionein *MT3* was post-transcriptionally down-regulated.

Changes in the abundance of histone-related proteins indicate that Mn deficiency also induces epigenetic modifications. For example, the H2A histone protein HTA10 showed increased abundance in Mn-deficient plants. While a specific function of HTA10 has not yet been inferred, it is of interest to note that the promoter activity of the coding gene was found to be sharply restricted to root cap cells in the root apical^25^. In contrast, the H2B protein HTB4 was less abundant when plants were grown on Mn-deplete media. The consequences of these changes in H2A/H2B dimer composition remain to be elucidated.

For the third and largest group of DE genes (605 genes; Supplemental Table S2), transcriptional changes were not translated into changes in protein abundance. This may be caused by a lack of reliable detection of the cognate protein or by a lack of detectable change in protein abundance. For approximately one-third of the transcripts in this group (218 mRNAs) proteins were identified and quantified, indicating that in a large fraction of the differentially expressed mRNAs transcriptional changes were disengaged from protein-level regulation. The most pronounced up-regulation was observed for the beta-glucosidase *BGLU23* (*PYK10*), a member of myrosinase protein family that cleaves the glucose group from glucosinolates. The remaining molecule is then converted to isothiocyanate, nitrile or thiocyanate, which are the active substances that function in defense responses to herbivores. Also, the gene encoding the PYK10-binding protein PBP1, an activator of BGLU23, was strongly up-regulated upon Mn deficiency. The most strongly down-regulated transcript was the glyceraldehyde-3-phosphate dehydrogenase subunit C2 (*GAPC2*); transcripts of the other subunit, *GAPA1*, showed also reduced abundance in Mn-deficient plants. Decreased transcripts were also observed for the sucrose synthases *SUS1* and *SUS4* and for several genes involved in photosynthesis. These changes indicate that the aerobic sucrose catabolic pathway is dramatically down-regulated when Mn is unavailable.

### Co-expression analysis reveals novel modules with putative functions in the manganese deficiency response

Functional information from differentially expressed genes can be inferred from the analysis of relationships of these genes (‘guilt by association’). To include the information of proteins that accumulate differentially upon Mn deficiency, a chimeric co-expression network was constructed from DE transcripts and DE proteins by using the MACCU software and publically available microarray experiments as a database^26^,^27^. Only DE genes that were co-expressed with a Pearson correlation coefficient greater than 0.75 were included in the network. This chimeric co-expression network comprises 366 DE transcripts and 129 DE proteins (with 33 of them regulated at both levels), which could be divided into several modules comprising transcripts/proteins of putatively related functions (Supplemental Table S3, Supplemental Fig. S1).

Sub-cluster C0 contains 105 transcripts and proteins related to photosynthesis, all of which were strongly down-regulated. At the protein level, the most strongly decreased abundance was observed for AtCg00710, a component of the photosystem II oxygen evolving core, PSBH. No corresponding transcript was detected for this protein. Due to the crucial function of Mn in PSII, we anticipated repression of genes related to photosynthesis upon Mn deficiency. It was, however, surprising that this response was evident in non-photosynthetic roots. It should be noted that the roots were exposed to light, resulting in the robust expression of PS genes in both leaves and roots, albeit with lower expression levels in the latter.

Another module in the co-expression network (C1) mainly contains down-regulated genes related to hypoxia such as *HEMOGLOBIN1* (*AHB1*) and 3 (*GLB3*), *ADH1*, the hypoxia-responsive transcription factors *ERF71* and *ERF73*, and genes encoding proteins involved in ethylene production such as *ACO1*. Also, the two sucrose synthase genes *SUS1* and *SUS4* were strongly down-regulated upon Mn deficiency.

**Figure 5:**
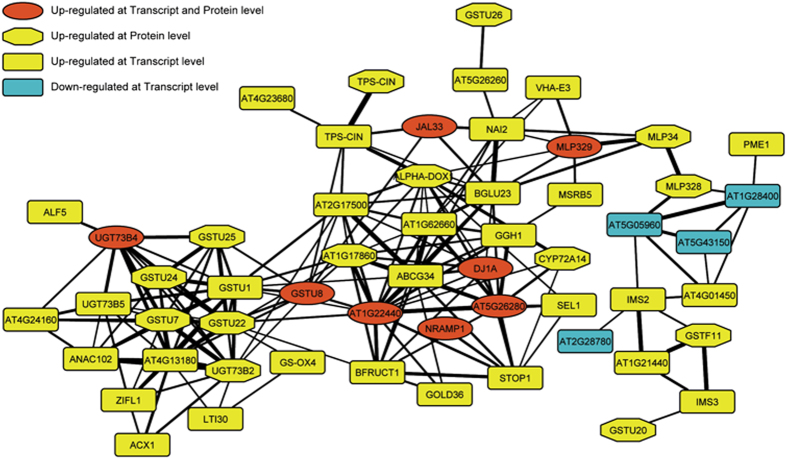
Central cluster of the chimeric co-expression network comprising Mn-responsive transcripts and proteins. The network was constructed with the MACCU toolkit comprising genes that were co-regulated with a Pearson correlation coefficient of *P* < 0.75. The weight of the edges is proportional to the *P*-value between the respective nodes.

**Figure 6:**
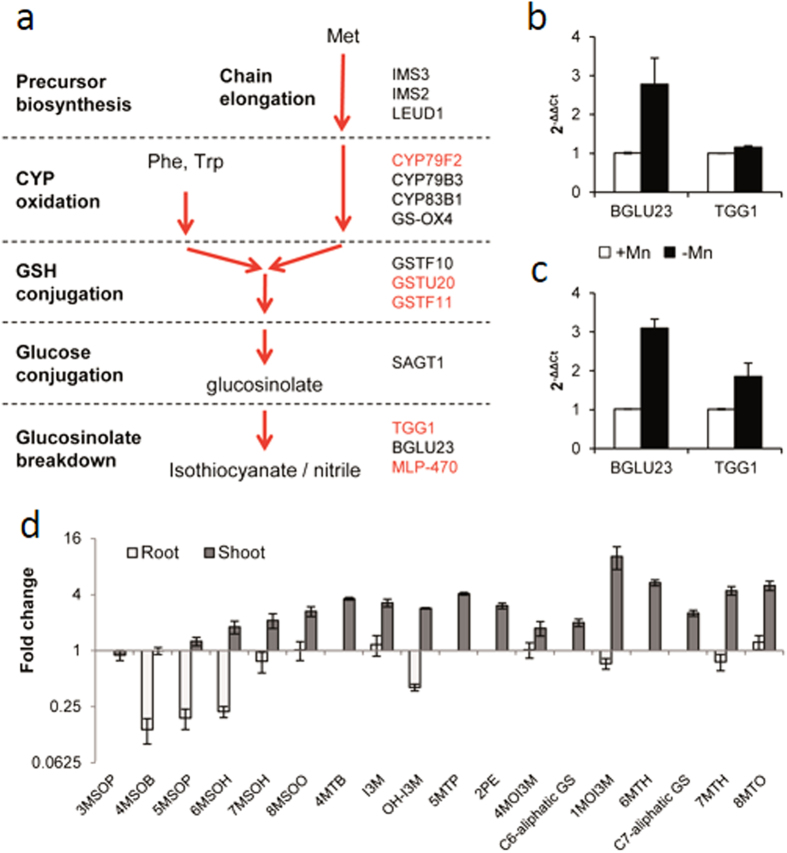
Glucosinolate biosynthesis in *Arabidopsis*. (**a**) RNAseq and iTRAQ results in the context of the glucosinolate biosynthesis pathway. Genes labeled in black and red increased significantly at the mRNA and protein level, respectively. (**b**,**c**) qRT-PCR results of *BGLU23* and *TGG1* expression levels in roots (**b**) and shoots (**c**). (**d**) UPLC analysis of glucosinolates in roots and leaves. Error bars represent standard deviations of three independent experiments.

The central part of the cluster C2 in this network comprises 56 nodes that mostly represent up-regulated genes (Fig. 5; Table 1). This cluster contains the Mn transporter *NRAMP1* and several transcripts/proteins related to glucosinolate metabolism that were all strongly up-regulated upon Mn deficiency, including *BGLU23*. To gain further insights into a putative involvement of glucosinolates in the Mn deficiency response, glucosinolate-related genes were surveyed among the DE genes. A metabolic pathway depicting the role of differentially expressed proteins/transcripts in glucosinolate biosynthesis and breakdown is shown in Fig. 6a, and expression changes are given in Table 1. For two of the genes encoding enzymes related to the breakdown of glucosinolates, *BGLU23* and *TGG1*, RNA-seq data were confirmed by qRT-PCR (Fig. 6b). Interestingly, several proteins that belong to families that participate in the pathway (*e.g.* glutathione transferases and glycosyl transferases) were found in this cluster, indicating a possible involvement of these transcripts/proteins in Mn deficiency-induced changes in glucosinolate metabolism.

### Metabolic analysis reveals dynamic changes in glucosinolate levels in response to Mn deficiency in leaves and roots

To investigate whether Mn deficiency alters glucosinolate biosynthesis, we analyzed the concentration of several major glucosinolates by UPLC-qTOF-MS in a targeted approach. Since glucosinolates can be synthesized in both roots and shoots^28^, the analysis was performed in both organs. In addition, we compared the induction of *BGLU23* and *TGG1* by Mn deficiency in roots with the expression changes in leaves *via* qRT-PCR. Similar to what has been observed for roots, the expression of both genes was found to be up-regulated in leaves (Fig. 6c). Unexpectedly, in roots, all glucosinolates under investigation showed either no change or decreases in their concentration. In particular, we observed strong decreases in the concentration of the aliphatic glucosinolates 4-, 5- and 6-methylsulfinylbutylglucosinolate (4MSOB, 5MSOP and 6MSOH) (Fig. 6d). In shoots, an opposite pattern was observed, with increases in most of the compounds under investigation (Fig. 6d), including aliphatic, aromatic and indolic glucosinolates. It should be noted that the compounds analyzed here are metabolic intermediates, and increased degradation of glucosinolates could lead to relative decreases in metabolites within the pathway. Rapid degradation, secretion of biologically active compounds, and/or export of glucosinolates to aboveground plant parts could be the reason for the lack of detectable increase in the surveyed glucosinolates in roots. A re-distribution of glucosinolates from roots to leaves would explain the similar changes in transcript levels of *BGLU23* and *TGG1* in the two organs, which stands in contrast to the observed changes in glucosinolate levels.

#### Deficiencies in Mn and Fe induces antagonistically-regulated processes

A comparison with previously conducted transcriptional and proteomic surveys on roots of Fe-deficient plants^28^,^29^ showed that several genes that are induced by Fe deficiency were down-regulated in roots of Mn-deficient plants. This subset includes genes involved in Fe mobilization such as the P-type ATPase *AHA2* or in the chelation of Fe (*NAS1*), and several genes encoding proteins with unknown function that were strongly induced by Fe deficiency. For example, *IRON-RESPONSIVE PROTEIN 3* (At2g14247), dramatically up-regulated in both roots and leaves of Fe-deficient plants^28^,^30^, was strongly down-regulated by Mn deficiency. Also up-regulated in response to Fe deficiency both at the transcript and protein level but down-regulated at the protein level Mn-deficient plants was the oxygenase superfamily protein At3g12900. At3g12900 is closely co-expressed with *IRT1* and the Myb-type transcription factors *MYB10* and *MYB72*, critical regulators of Fe uptake from alkaline soils that limit the mobility of Fe^31^, which may indicate a role of At3g12900 in Fe acquisition from Fe pools of low availability.

Manganese deficiency was shown to increase root hair length and density, probably to compensate for the low mobility of Mn^15^,^32^. Several genes with putative or validated roles in root hair development showed increased expression under Mn-deficient conditions but decreased expression in Fe-deficient plants (*DER1*, *AGP14*, *FLA9*). AGP14 was previously associated with root hair elongation^26^ and may be critical in the formation of longer root hairs under Mn-deficient conditions. Iron deficiency does not increase root hair length^33^.

The most highly induced gene in Mn-deficient plants, *BGLU23* (*PYK10*), was the most down-regulated gene in Fe-deficient plants. Also the expression of a partner of BGLU23, *PBP1*, followed a similar pattern. Moreover, three genes involved in glucosinolate breakdown, (*CWINV1*, At1g62660 and the nitrile specifier *NSP1*), the glucosinolate biosynthesis gene *MAM3,* and the plasma membrane-localized transporter ABCG34, were oppositely regulated by Mn and Fe. Together these data suggest that the acquisition responses of both nutrients are controlled in an antagonistic manner.

## Discussion

Despite the importance of Mn for plant growth, limited information is available regarding the proteins involved in the acquisition of Mn from the soil solution and the mechanisms by which plants re-calibrate cellular Mn homeostasis. Also, the signaling cascades that control these processes await elucidation. Several transporters can catalyze the movement of Mn across membranes^14^,^34^, but except for the transporters of the Nramp family (AtNRAMP1, 3 and 4 in *Arabidopsis*;^7^,^9^,^10^) little is known regarding their physiological relevance for cellular Mn homeostasis under conditions of low Mn availability. In the present investigation, NRAMP1 was found to be differentially expressed between Mn-sufficient and Mn-deficient plants at both the transcript and protein level, supporting its critical importance for Mn uptake. Induction of *NRAMP1* upon Mn deficiency was moderate though and not comparable with the dramatic increase in *IRT1* transcript abundance upon Fe deficiency. Also of note, pronounced changes in Mn efficiency between barley genotypes were not evident in solution culture^35^, indicating that other factors such as root-soil or root-microbe interaction dictate Mn uptake rate from pools of low phyto-availability and thus Mn efficiency.

### Concordance of mRNA and Protein Expression

Regulation of gene expression is largely decoupled from protein dynamics, a phenomenon that has been observed through all types of organisms, including plants^16^. Several attempts have been undertaken to estimate how much of the changes observed at the nucleotide level are translated into proteins^36^,^37^. These efforts were complicated by several factors. Firstly, in many cases transcriptomic and proteomic profiling was carried out in different laboratories, introducing a substantial bias by variations in experimental conditions. A second technical bias is introduced by the fundamental differences in extraction and detection of transcripts and proteins, which differ in their levels of sensitivity and measurement error. Moreover, proteomics have lagged behind transcriptomics in terms of coverage, leaving the majority of identified mRNAs of protein coding genes without cognate proteins in most surveys. Finally, a highly dynamic interplay between mRNA and proteins complicates all predictions from one level to another. In the present study, only for 14% of the differentially expressed proteins associated transcripts were detected that were regulated in the same direction. Considering the correlation of mRNA and protein abundance for those proteins that were detected as cognate pairs, an overall concordance of the two layers of approximately 10% can be estimated, a value that matched that estimated in a recent study on Fe-deficient plants^29^.

Unexpectedly, from the three groups of differentially regulated genes, the group that we defined as ‘robustly regulated by Mn’ was the smallest (48 transcripts/proteins). The second group, comprising chiefly post-transcriptionally regulated proteins, was not only much bigger (296 proteins), but also contained proteins that showed more pronounced changes in expression ratio. While the reason(s) for this observation cannot be deduced from the data at hand, it is of interest to note that in this group proteins with functions in translation, mRNA processing, and histone modifications were overrepresented, indicative of a largely unexplored regulatory layer that may have a strong impact on Mn homeostasis. This (transcriptionally) hidden layer may affect the abundance of proteins independent of their transcriptional level, thereby contributing to the gap between mRNA and protein abundance.

The largest group comprised genes that were solely transcriptionally regulated (605 genes). Included in this subset are: firstly, genes with noisy expression that might be beneficial in terms of system stabilization and plant fitness^38^; secondly, genes for which the detection or quantification of the cognate protein was obscured for technical reasons, and, thirdly, transcripts for which the cognate protein is unstable or inefficiently translated, necessitating a pronounced regulation at the transcriptional level to stabilize the proteomic readout. While a general conclusion about the relative physiological importance of these groups is difficult to be drawn from the current data set, it becomes obvious that transcription is intrinsically stochastic and information regarding the transcripts and proteins involved in a given process and their regulatory patterns can only be achieved by an integrative approach accessing different levels of gene activity. Also, it appears that only a relatively small part of Mn deficiency-induced changes in gene expression can be attributed to a regulation at the transcriptional level.

#### Production of glucosinolates: an integral part of the Mn acquisition strategy?

Glucosinolates are a class of sulfur- and nitrogen-containing organic compounds derived from glucose and amino acids, which are involved in plant defense. Glucosinolates are formed by an aminoacidic origin radical and a glucose moiety, linked by a thioesther bond. They occur as secondary metabolites in almost all species of the order Brassicales. The reasons for the strong regulation of glucosinolate-associated genes is not entirely clear, but the pronounced changes suggest auxiliary roles of glucosinolates in the Mn deficiency response. In roots, genes involved in the biosynthesis and degradation of glucosinolates were found to be up-regulated at the mRNA and protein level. Mn deficiency-induced up-regulation of two genes (out of two tested), *BGLU23* and *TGG1*, was also observed in leaves, which stands in contrast to the opposite pattern of glucosinolate accumulation in the two organs. Thus, the increase in glucosinolates in leaves might have partly occurred at the expense of roots, which could export these substances to the aboveground plant parts. Glucosinolates are important for the defense against pathogens and innate immunity^39^,^40^,^41^. It is thus tempting to assume that the increase in the production of glucosinolates and their breakdown products is an adaptive response triggered by increased pathogen susceptibility under conditions of Mn deficiency. This stress-induced decrease in pathogen resistance might be further enhanced by the down-regulation of Fe-responsive genes, in particular those that are related to the production of phenolics, which may lead to impaired lignification^42^ and, as a consequence, increased disease susceptibility. This supposition is supported by the down-regulation of genes that are important for the uptake of Fe at alkaline pH, which is mainly mediated via the excretion of phenolic compounds^30^,^43^,^44^. While the precise role of glucosinolates needs further elucidation, we suppose that reprograming of glucosinolate metabolism in roots and leaves represents an important and integral part of the response of *Arabidopsis* to Mn deficiency. A plausible alternative function of glucosinolates could lie in the interaction with the rizhosphere microbiome as a chemotoxine to inhibit bacterial growth and eliminate bacterial competence for available Mn.

## Conclusions

In summary, we here provide a near complete inventory of transcripts and proteins that are responsive to Mn. This catalog reveals that several regulatory processes are engaged by Mn deficiency, including changes in chromatin structure, transcription, transcript maturation and stability, as well as alterations in translation efficiency and protein stability. By comparing transcriptomic, proteomic and metabolomic data, we discovered Mn deficiency-induced changes that are hidden when only in one of these levels is interrogated. Our data further reveal massive Mn-specific changes in metabolism, which contrast similar processes responsive to Festarvation.

## Additional Information

**How to cite this article**: Rodríguez-Celma, J. *et al*. Systems-wide analysis of manganese deficiency-induced changes in gene activity of *Arabidopsis* roots. *Sci. Rep.* **6**, 35846; doi: 10.1038/srep35846 (2016).

**Publisher’s note:** Springer Nature remains neutral with regard to jurisdictional claims in published maps and institutional affiliations.

## Supplementary Material

Supplementary Information

Supplementary Table S1

Supplementary Table S2

Supplementary Table S3

## Figures and Tables

**Table 1:** Expression changes of genes and proteins mentioned in the text and/or displayed in the figures.

Gene ID	Symbol	Description	ΔRPKM (−Mn/+Mn)	iTRAQ (−Mn/+Mn)	Pathway
*Glucosinolate metabolism*					
AT4G39940	AKN2	APS-kinase 2	**21.31**	1.13	Sulphur metabolism
AT1G18570	MYB51	Myb domain protein 51	**20.34**		Transcription factor
AT5G23010	IMS3	Methylthioalkylmalate synthase 1	**46.67**	1.32	Chain Elongation
AT5G23020	IMS2	2-isopropylmalate synthase 2	**102.33**	1.39	Chain Elongation
AT2G43100	LEUD1	Isopropylmalate isomerase 2	**30.01**	0.93	Chain Elongation
AT1G16400	CYP79F2	Cytochrome P450, family 79, subfamily F, polypeptide 2	19.99	**1.52**	CYP oxidation
AT1G62570	GS-OX4	Flavin-monooxygenase glucosinolate S-oxygenase 4	**17.9**	0.98	CYP oxidation
AT2G22330	CYP79B3	Cytochrome P450, family 79, subfamily B, polypeptide 3	**22.41**		CYP oxidation
AT4G31500	CYP83B1	Cytochrome P450, family 83, subfamily B, polypeptide 1	**62.13**	1.34	CYP oxidation
AT1G78370	GSTU20	Glutathione S-transferase TAU 20		**1.76**	GSH conjugation
AT2G30870	GSTF10	Glutathione S-transferase PHI 10	**141.57**	1.31	GSH conjugation
AT3G03190	GSTF11	Glutathione S-transferase F11		**1.47**	GSH conjugation
AT2G43820	SAGT1	UDP-glucosyltransferase 74F2		**1.62**	Glucose conjugation
AT5G26000	TGG1	Thioglucoside glucohydrolase 1		**1.56**	Breakdown
AT3G09260	BGLU23	Glycosyl hydrolase superfamily protein	**935.1**	1.13	Breakdown
AT3G16400	MLP-470	Nitrile specifier protein 1		**1.68**	Breakdown
*Photosyntesis*					
AT1G15820	CP24	Light harvesting complex photosystem II subunit 6	**−48.07**	1.03	LHC II
AT1G29910	AB180	Chlorophyll A/B binding protein 3	**−44.57**	**0.62**	LHC II
AT1G29930	AB140	Chlorophyll A/B binding protein 1	**−755.74**		LHC II
AT2G05070	LHCB2	Photosystem II light harvesting complex gene 2.2	**−36.05**		LHC II
AT2G05100	LHCB2	Photosystem II light harvesting complex gene 2.1	**−39.79**	0.87	LHC II
AT2G34420	LHB1B2	Photosystem II light harvesting complex gene B1B2	**−154.71**	0.73	LHC II
AT3G08940	LHCB4.2	Light harvesting complex photosystem II	**−52.46**	0.96	LHC II
AT4G10340	LHCB5	Light harvesting complex of photosystem II 5	**−136.41**	0.90	LHC II
AT5G01530	LHCB4.1	Light harvesting complex photosystem II	**−78.17**	1.04	LHC II
AT5G54270	LHCB3	Light-harvesting chlorophyll B-binding protein 3	**−75.34**	0.95	LHC II
AT1G03600	PSB27	Photosystem II family protein	**−39.8**	0.79	PS II
AT1G06680	OE23	Photosystem II subunit P-1	**−91.79**	0.76	PS II
AT1G44575	CP22	Chlorophyll A-B binding family protein	**−30.84**	1.04	PS II
AT1G51400	NFU1	Photosystem II 5 kD protein	**−22.61**	0.80	PS II
AT1G67740	PSBY	Photosystem II BY	**−29.75**		PS II
AT2G06520	PSBX	Photosystem II subunit X	**−77.31**		PS II
AT2G30570	PSBW	Photosystem II reaction center W	**−62.72**		PS II
AT3G21055	PSBTN	Photosystem II subunit T	**−45.86**	0.74	PS II
AT3G50820	OEC33	Photosystem II subunit O-2	**−36.6**	**0.7**	PS II
AT4G05180	PSBQ	Photosystem II subunit Q-2	**−36.54**	**0.66**	PS II
AT4G21280	PSBQ	Photosystem II subunit QA	**−42.95**	0.79	PS II
AT5G66570	MSP-1	Photosystem II oxygen-evolving complex 1	**−133.64**	0.77	PS II
ATCG00710	PSBH	Photosystem II reaction center protein H		**0.5**	PS II
AT2G26500	UGT76D1	Cytochrome b6f complex subunit (petM), putative	**−49.41**		Redox chain
AT4G03280	PETC	Photosynthetic electron transfer C	**−39.14**	0.74	Redox chain
AT1G20340	DRT112	Cupredoxin superfamily protein	**−58.42**	0.81	Redox chain
AT4G04640	PC1	ATPase, F1 complex, gamma subunit protein	**−19.75**	1.35	Redox chain
AT4G09650	PD	ATP synthase delta-subunit gene	**−16.51**	0.90	Redox chain
AT4G32260	PDE334	ATPase, F0 complex, subunit bacterial/chloroplast	**−30.64**	0.91	Redox chain
AT1G03130	PSAD-2	Photosystem I subunit D-2	**−16.58**	0.75	PS I
AT1G08380	PSAO	Photosystem I subunit O	**−67.2**	0.95	PS I
AT1G30380	PSAK	Photosystem I subunit K	**−31.53**	**0.72**	PS I
AT1G31330	PSAF	Photosystem I subunit F	**−67.05**	0.85	PS I
AT1G52230	PSAH-2	Photosystem I subunit H2	**−20.98**	0.72	PS I
AT1G55670	PSAG	Photosystem I subunit G	**−83.33**	0.99	PS I
AT2G20260	PSAE-2	Photosystem I subunit E-2	**−27.77**	0.81	PS I
AT2G46820	PSAP	Photosystem I P subunit	**−16.9**	1.02	PS I
AT3G16140	PSAH-1	Photosystem I subunit H-1	**−32.9**	0.87	PS I
AT4G02770	PSAD-1	Photosystem I subunit D-1	**−36.21**	**0.73**	PS I
AT4G12800	PSAL	Photosystem I subunit l	**−61.76**	0.84	PS I
AT4G28750	PSAE-1	Photosystem I reaction centre subunit IV/PsaE	**−34.63**	**0.66**	PS I
AT5G64040	PSAN	Photosystem I reaction center subunit PSI-N, chloroplast, putative/PSI-N, putative	**−62.37**	**0.73**	PS I
AT1G61520	LHCA3	Photosystem I light harvesting complex gene 3	**−56.35**	0.81	LHC I
AT3G47470	CAB4	Light-harvesting chlorophyll-protein complex I subunit A4	**−113**	0.93	LHC I
AT3G54890	LHCA1	Photosystem I light harvesting complex gene 1	**−53.58**	0.91	LHC I
AT3G61470	LHCA2	Photosystem I light harvesting complex gene 2	**−84.36**	0.89	LHC I
AT1G60950	FD2	2Fe-2S ferredoxin-like superfamily protein	**−44.5**	0.91	Final
AT5G66190	LFNR1	Ferredoxin-NADP(+)-oxidoreductase 1	**−26.21**	1.20	Final
AT1G32060	PRK	Phosphoribulokinase	**−34.94**	0.95	Calvin cycle
AT1G67090	RBCS1A	Ribulose bisphosphate carboxylase small chain 1A	**−341.12**	0.85	Calvin cycle
AT5G38410	LCR80	Ribulose bisphosphate carboxylase (small chain)	**−59.24**		Calvin cycle
AT2G39730	RCA	Rubisco activase	**−87.93**	0.88	Calvin cycle
AT1G12900	GAPA-2	Glyceraldehyde 3-phosphate dehydrogenase A subunit 2	**−37.94**	1.01	Calvin cycle
AT1G42970	GAPB	Glyceraldehyde-3-phosphate dehydrogenase B subunit	**−33.63**	1.04	Calvin cycle
AT3G26650	GAPA	Glyceraldehyde 3-phosphate dehydrogenase A subunit	**−98.66**	1.31	Calvin cycle
AT2G01140	PDE345	Aldolase superfamily protein	**−59.01**	0.91	Calvin cycle
AT2G21330	FBA1	Fructose-bisphosphate aldolase 1	**−27.05**	0.81	Calvin cycle
AT4G38970	FBA2	Fructose-bisphosphate aldolase 2	**−49.19**	0.89	Calvin cycle
AT3G60750	CHO	Transketolase	**−52.41**	1.28	Calvin cycle
AT5G61410	EMB2728	D-ribulose-5-phosphate-3-epimerase	**−22.8**	0.95	Calvin cycle
*Central metabolism*					
AT4G26270	PFK3	Phosphofructokinase 3	**−21.65**	0.98	Glycolysis
AT2G36460	FBA6	Aldolase superfamily protein	**−197.05**	0.73	Glycolysis
AT2G21170	TIM	Triosephosphate isomerase	**−38.87**	0.95	Glycolysis
AT1G13440	GAPC2	Glyceraldehyde-3-phosphate dehydrogenase C2	**−1286.09**	0.94	Glycolysis
AT3G12780	PGK1	Phosphoglycerate kinase 1	**−23.93**	0.93	Glycolysis
AT2G36530	LOS2	Enolase	**−89.97**	0.89	Glycolysis
AT2G36580		Pyruvate kinase family protein	**−95.38**	1.22	Glycolysis
AT2G44350	CSY4	Citrate synthase family protein	**18.94**	1.04	TCA cycle
AT1G65930	CICDH	Cytosolic NADP + -dependent isocitrate dehydrogenase		**1.42**	TCA cycle
AT5G08530	CI51	51 kDa subunit of complex I	**−21.11**	0.99	Respiration
AT3G22370	AOX1A	Alternative oxidase 1A	**16.86**	1.10	Respiration
AT4G17260		Lactate/malate dehydrogenase family protein	**−43.97**	0.78	Fermentation
AT1G77120	ADH1	Alcohol dehydrogenase 1	**−1957.5**	0.55	Fermentation
AT4G33070	PDC1	Thiamine pyrophosphate dependent pyruvate decarboxylase family protein	**−707.92**	0.65	Fermentation
AT5G66985		Unknown	**−187.13**	0.81	Fermentation
AT5G15120	PCO1	Protein of unknown function (DUF1637)	**−60.33**	0.74	Fermentation
*Cluster C2(* Fig. 5)					
AT1G80830	NRAMP1	Natural resistance-associated macrophage protein 1	**27.73**	**1.51**	
AT3G25820	TPS-CIN	Terpene synthase-like sequence-1,8-cineole	5.64	**2.11**	
AT4G34135	UGT73B2	UDP-glucosyltransferase 73B2		**1.83**	
AT1G78340	GSTU22	Glutathione S-transferase TAU 22		**1.69**	
AT2G01520	MLP328	MLP-like protein 328		**1.67**	
AT1G17180	GSTU25	Glutathione S-transferase TAU 25	22.59	**1.64**	
AT3G01420	DOX1	Peroxidase superfamily protein		**1.62**	
AT1G17170	GSTU24	Glutathione S-transferase TAU 24	24.51	**1.56**	
AT2G01530	MLP329	MLP-like protein 329	243.22	**1.56**	
AT1G70850	MLP34	MLP-like protein 34		**1.51**	
AT1G22440		Zinc-binding alcohol dehydrogenase family protein	21.43	**1.49**	
AT2G15490	UGT73B4	UDP-glycosyltransferase 73B4	23.43	**1.48**	
AT3G09270	GSTU8	Glutathione S-transferase TAU 8	66.19	**1.48**	
AT3G14990	DJ1A	Class I glutamine amidotransferase-like superfamily protein	341.33	**1.48**	
AT1G17190	GSTU26	Glutathione S-transferase tau 26	10.53	**1.46**	
AT3G14680	CYP72A14	Cytochrome P450, family 72, subfamily A, polypeptide 14		**1.43**	
AT5G26280		TRAF-like family protein	133.09	**1.41**	
AT2G29420	GSTU7	Glutathione S-transferase tau 7		**1.41**	
AT3G16450	JAL33	Mannose-binding lectin superfamily protein	228.35	**1.4**	
AT1G17860		Kunitz family trypsin and protease inhibitor protein	40.72	**1.4**	
AT3G25830	TPS-CIN	Terpene synthase-like sequence-1,8-cineole		**97.45**	
AT3G15950	NAI2	DNA topoisomerase-related	**81.69**	1.20	
AT4G01450		Nodulin MtN21/EamA-like transporter		**81.41**	
AT4G04830	MSRB5	Methionine sulfoxide reductase B5	**72.38**	1.01	
AT3G50970	LTI30	Dehydrin family protein	**71.95**	1.21	
AT4G13180		NAD(P)-binding Rossmann-fold	**54.24**	1.07	
AT5G63790	ANAC102	NAC domain containing protein 102	**53.02**		
AT2G17500		Auxin efflux carrier family protein	**41.68**		
AT1G64200	VHA-E3	Vacuolar H + -ATPase subunit E isoform 3	**37.23**	1.18	
AT2G29490	GSTU1	Glutathione S-transferase TAU 1	**37.14**	1.34	
AT5G26260		TRAF-like family protein	**36.24**	1.18	
AT3G13790	BFRUCT1	Glycosyl hydrolases family 32 protein	**35.46**	1.07	
AT1G78000	SEL1	Sulfate transporter 1;2	**34.83**	1.13	
AT4G23680		Polyketide cyclase/dehydrase and lipid transport	**34.68**	1.36	
AT1G62660		Glycosyl hydrolases family 32 protein	**34.23**	1.27	
AT1G21440		Phosphoenolpyruvate carboxylase family protein	**33.73**	1.23	
AT2G36380	ABCG34	Pleiotropic drug resistance 6	**29.82**	1.25	
AT5G13750	ZIFL1	Zinc induced facilitator-like 1	**29.66**		
AT1G78660	GGH1	Gamma-glutamyl hydrolase 1	**26.72**	1.13	
AT4G24160		Alpha/beta-Hydrolases superfamily protein	**23.28**	1.23	
AT4G16760	ACX1	Acyl-CoA oxidase 1	**19.87**	1.25	
AT4G12390	PME1	Pectin methylesterase inhibitor 1	**19.3**	1.08	
AT1G34370	STOP1	C2H2 and C2HC zinc fingers superfamily protein	**18.68**		
AT1G54030	GOLD36	GDSL-like Lipase/Acylhydrolase superfamily protein	**16.92**	1.11	
AT3G23560	ALF5	MATE efflux family protein	**16.9**		
AT2G15480	UGT73B5	UDP-glucosyl transferase 73B5	**16.38**	1.18	
AT2G28780		Unknown	**−30.55**		
AT5G43150		Unknown	**−43.56**		
AT1G28400		Unknown	**−119.83**	0.80	
AT5G05960		Bifunctional inhibitor/lipid-transfer protein/seed storage 2S albumin superfamily protein	**−168.75**	0.93	
*Robustly Mn-responsive genes (i.e. changed at the mRNA and protein level) with miscellaneous functions*					
AT4G15610		Uncharacterised protein family (UPF0497)	**93.23**	**1.78**	
AT1G18670	IBS1	Protein kinase superfamily protein	**−21.49**	**1.58**	
AT5G42250		Zinc-binding alcohol dehydrogenase family protein	**21.69**	**1.58**	
AT1G45145	H5	Thioredoxin H-type 5	**107.51**	**1.5**	
AT3G26450		Polyketide cyclase/dehydrase and lipid transport superfamily protein	**22.43**	**1.5**	
AT5G60660	PIP2;4	Plasma membrane intrinsic protein 2;4	**21.92**	**1.5**	
AT2G45960	HH2	Plasma membrane intrinsic protein 1B	**353.05**	**1.49**	
AT5G40890	CLC-A	Chloride channel A	**17.06**	**1.49**	
AT5G14120		Major facilitator superfamily protein	**53.91**	**1.46**	
AT4G30020		PA-domain containing subtilase family protein	**−36.43**	**0.74**	
AT2G28950	EXP6	Pxpansin A6	**−22.64**	**0.73**	
AT3G11930		Adenine nucleotide alpha hydrolases-like superfamily protein	**−102.64**	**0.73**	
AT3G15650		alpha/beta-Hydrolases superfamily protein	**−20.76**	**0.72**	
AT4G01630	EXP17	Expansin A17	**−33.42**	**0.72**	
AT2G16060	AHB1	Hemoglobin 1	**−894.75**	**0.7**	
AT1G03820		Unknown	**−23.67**	**0.69**	
AT4G12880	ENODL19	Early nodulin-like protein 19	**−38.37**	**0.69**	
AT5G19550	AAT2	Aspartate aminotransferase 2	**−341.57**	**0.69**	
AT1G69230	SP1L2	SPIRAL1-like2	**−51.23**	**0.68**	
AT5G15970	Cor6.6	Stress-responsive protein (KIN2)/stress-induced protein (KIN2)/cold-responsive protein (COR6.6)/cold-regulated protein (COR6.6)	**−147.81**	**0.68**	
AT2G38530	cdf3	Lipid transfer protein 2	**−55.52**	**0.66**	
AT1G09750		Eukaryotic aspartyl protease family protein	**−18.27**	**0.64**	
AT5G02380	MT2B	Metallothionein 2B	**−204.51**	**0.64**	
AT5G15960	KIN1	Stress-responsive protein (KIN1)/stress-induced protein (KIN1)	**−64.62**	**0.63**	
AT5G38980		Unknown	**−18.37**	**0.62**	
AT4G27450		Aluminium induced protein with YGL and LRDR motifs	**−1161.92**	**0.61**	
AT2G46630		Unknown	**−16.73**	**0.6**	
AT3G03270		Adenine nucleotide alpha hydrolases-like superfamily protein	**−490.84**	**0.51**	
AT2G17850		Rhodanese/Cell cycle control phosphatase superfamily protein	**−279.68**	**0.41**	

Numbers represent log2 values. Bold letters indicate statistically significant changes.
