# Supplementary material for: Systems-wide analysis of manganese deficiency-induced changes in gene activity of Arabidopsis roots
Source: Sci Rep. 2016 Nov 2;6:35846. doi: 10.1038/srep35846 (PMC5090222; doi:10.1038/srep35846)
Supplement: Supplementary Information [file srep35846-s1.doc]

**ONLINE DATA SUPPLEMENT**

**Systems-wide analysis of manganese deficiency-induced changes in gene activity of *Arabidopsis* roots**

Jorge Rodríguez-Celma^1^, Yi-Hsiu Tsai^1^, Tuan-Nan Wen^1^, Yu-Ching Wu^1^, Catherine Curie^2^ and Wolfgang Schmidt^1,3,4,5^

^1^Institute of Plant and Microbial Biology, Academia Sinica, 128 Academia Road, Taipei, Taiwan

^2^Biochimie et Physiologie Moléculaire des Plantes, Centre National de la Recherche Scientifique, Institut National pour la Recherche Agronomique, Laboratoire de Biochimie et Physiologie Moléculaire des Plantes, INRA/SupAgro, Université Montpellier 2, Montpellier, France

^3^Graduate Institute of Biotechnology, National Chung Hsing University, Taichung, Taiwan

^4^Genome and Systems Biology Degree Program, College of Life Science, National Taiwan University, Taipei, Taiwan

^5^Corresponding author: Wolfgang Schmidt; e-mail: wosh@gate.sinica.edu.tw

Institute of Plant and Microbial Biology, Academia Sinica, Taipei, Taiwan

Phone: +886-02-27871038

Fax: +886-02-2787954

**Supplemental data set S1.** Annotated spectra of proteins and peptides identified in roots and leaves (available at ProteomeXchange Consortium; dataset identifier PXD003309).

**Supplemental Figure S1.** Chimeric mRNA/protein co-expression network.

**Supplemental Figure S2.** Mn deficiency-induced changes in the concentration of major glucosinolates.

**Supplemental Table S1.** RNA-seq data iTRAQ ratios and protein/peptide identification information.

**Supplemental Table S2.** Transcriptionally and protein-level regulated genes.

**Supplemental Table S3.** Genes comprising the three sub-clusters in the co-expression network.
