# Supplementary material for: Systems-wide analysis of manganese deficiency-induced changes in gene activity of Arabidopsis roots
Source: Sci Rep. 2016 Nov 2;6:35846. doi: 10.1038/srep35846 (PMC5090222; doi:10.1038/srep35846)
Supplement: Supplementary Table S1 [file srep35846-s2.doc]

ONLINE DATA SUPPLEMENT

**Systems-wide analysis of manganese deficiency-induced changes in gene activity of *Arabidopsis* roots**

Jorge Rodríguez-Celma^1^, Yi-Hsiu Tsai^1^, Tuan-Nan Wen^1^, Yu-Ching Wu^1^, Catherine Curie^2^ and Wolfgang Schmidt^1,3,4,5^

^1^Institute of Plant and Microbial Biology, Academia Sinica, 128 Academia Road, Taipei, Taiwan

^2^Biochimie et Physiologie Moléculaire des Plantes, Centre National de la Recherche Scientifique, Institut National pour la Recherche Agronomique, Laboratoire de Biochimie et Physiologie Moléculaire des Plantes, INRA/SupAgro, Université Montpellier 2, Montpellier, France

^3^Graduate Institute of Biotechnology, National Chung Hsing University, Taichung, Taiwan

^4^Genome and Systems Biology Degree Program, College of Life Science, National Taiwan University, Taipei, Taiwan

^5^Corresponding author: Wolfgang Schmidt; e-mail: wosh@gate.sinica.edu.tw

Institute of Plant and Microbial Biology, Academia Sinica, Taipei, Taiwan

Phone: +886-02-27871038

Fax: +886-02-2787954

Supplementary Figures S1 and S2, Supplemental Table 2


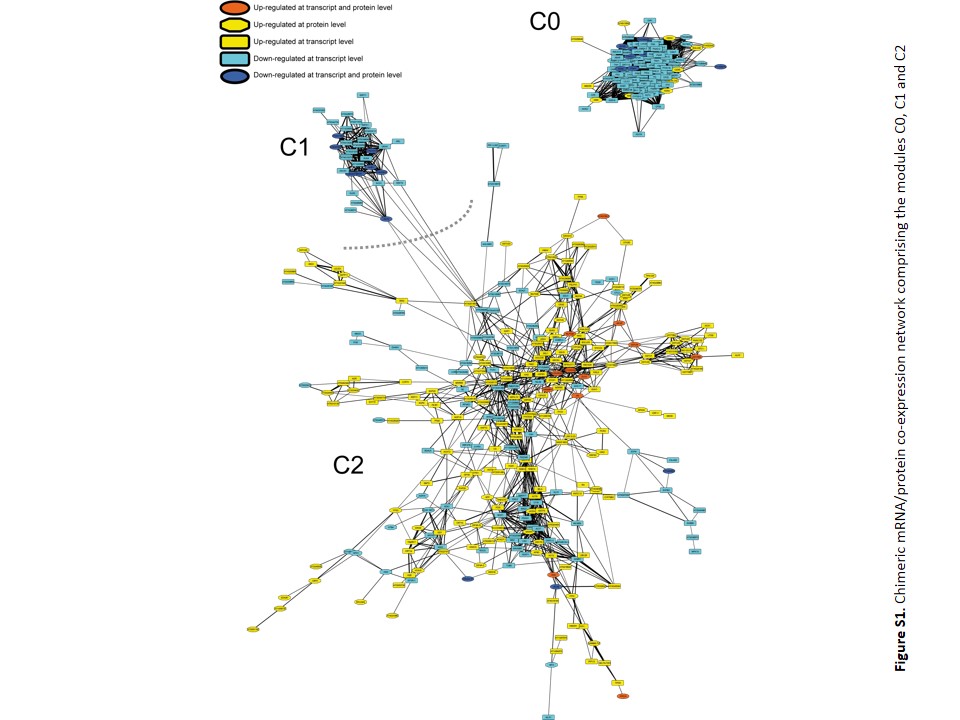


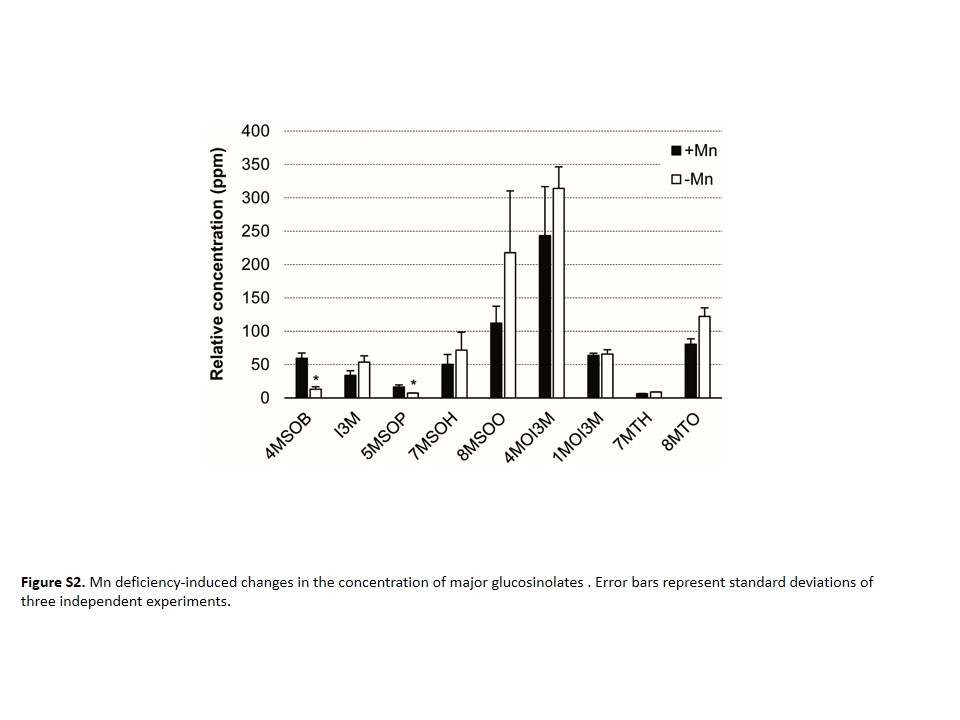


**Table S2. Transcriptionally and protein-level regulated genes.**

| **#GeneID** | **Symbol** | **Description** | **ΔRPKM** | **iTRAQ ratio** |
| --- | --- | --- | --- | --- |
| *Group 1: Genes with simultaneous change in transcript and protein abundance (robustly Mn-regulated)* | | | | |
| AT4G15610 |  | Uncharacterised protein family (UPF0497) | **93.23** | **1.78** |
| AT1G18670 | IBS1 | Protein kinase superfamily protein | **-21.49** | **1.58** |
| AT5G42250 |  | Zinc-binding alcohol dehydrogenase family protein | **21.69** | **1.58** |
| AT2G01530 | MLP329 | MLP-like protein 329 | **243.22** | **1.56** |
| AT1G80830 | NRAMP1 | natural resistance-associated macrophage protein 1 | **27.73** | **1.51** |
| AT1G45145 | H5 | thioredoxin H-type 5 | **107.51** | **1.5** |
| AT3G26450 |  | Polyketide cyclase/dehydrase and lipid transport superfamily protein | **22.43** | **1.5** |
| AT5G60660 | PIP2;4 | plasma membrane intrinsic protein 2;4 | **21.92** | **1.5** |
| AT1G22440 |  | Zinc-binding alcohol dehydrogenase family protein | **21.43** | **1.49** |
| AT2G45960 | HH2 | plasma membrane intrinsic protein 1B | **353.05** | **1.49** |
| AT5G40890 | CLC-A | chloride channel A | **17.06** | **1.49** |
| AT2G15490 | UGT73B4 | UDP-glycosyltransferase 73B4 | **23.43** | **1.48** |
| AT3G09270 | GSTU8 | glutathione S-transferase TAU 8 | **66.19** | **1.48** |
| AT3G14990 | DJ1A | Class I glutamine amidotransferase-like superfamily protein | **341.33** | **1.48** |
| AT5G14120 |  | Major facilitator superfamily protein | **53.91** | **1.46** |
| AT5G26280 |  | TRAF-like family protein | **133.09** | **1.41** |
| AT3G16450 | JAL33 | Mannose-binding lectin superfamily protein | **228.35** | **1.4** |
| AT4G30020 |  | PA-domain containing subtilase family protein | **-36.43** | **0.74** |
| AT5G15120 |  | Protein of unknown function (DUF1637) | **-60.33** | **0.74** |
| AT2G28950 | EXP6 | expansin A6 | **-22.64** | **0.73** |
| AT2G36460 |  | Aldolase superfamily protein | **-197.05** | **0.73** |
| AT3G11930 |  | Adenine nucleotide alpha hydrolases-like superfamily protein | **-102.64** | **0.73** |
| AT4G02770 | PSAD-1 | photosystem I subunit D-1 | **-36.21** | **0.73** |
| AT5G64040 | PSAN | photosystem I reaction center subunit PSI-N, chloroplast, putative / PSI-N, putative (PSAN) | **-62.37** | **0.73** |
| AT1G30380 | PSAK | photosystem I subunit K | **-31.53** | **0.72** |
| AT3G15650 |  | alpha/beta-Hydrolases superfamily protein | **-20.76** | **0.72** |
| AT4G01630 | EXP17 | expansin A17 | **-33.42** | **0.72** |
| AT2G16060 | AHB1 | hemoglobin 1 | **-894.75** | **0.7** |
| AT3G50820 | OEC33 | photosystem II subunit O-2 | **-36.6** | **0.7** |
| AT1G03820 |  | Unknown | **-23.67** | **0.69** |
| AT4G12880 | ENODL19 | early nodulin-like protein 19 | **-38.37** | **0.69** |
| AT5G19550 | AAT2 | aspartate aminotransferase 2 | **-341.57** | **0.69** |
| AT1G69230 | SP1L2 | SPIRAL1-like2 | **-51.23** | **0.68** |
| AT5G15970 | Cor6.6 | stress-responsive protein (KIN2) / stress-induced protein (KIN2) / cold-responsive protein (COR6.6) / cold-regulated protein (COR6.6) | **-147.81** | **0.68** |
| AT2G38530 | cdf3 | lipid transfer protein 2 | **-55.52** | **0.66** |
| AT4G05180 | PSBQ | photosystem II subunit Q-2 | **-36.54** | **0.66** |
| AT4G28750 | PSAE-1 | Photosystem I reaction centre subunit IV / PsaE protein | **-34.63** | **0.66** |
| AT4G33070 |  | Thiamine pyrophosphate dependent pyruvate decarboxylase family protein | **-707.92** | **0.65** |
| AT1G09750 |  | Eukaryotic aspartyl protease family protein | **-18.27** | **0.64** |
| AT5G02380 | MT2B | metallothionein 2B | **-204.51** | **0.64** |
| AT5G15960 | KIN1 | stress-responsive protein (KIN1) / stress-induced protein (KIN1) | **-64.62** | **0.63** |
| AT1G29910 | AB180 | chlorophyll A/B binding protein 3 | **-44.57** | **0.62** |
| AT5G38980 |  | Unknown | **-18.37** | **0.62** |
| AT4G27450 |  | Aluminium induced protein with YGL and LRDR motifs | **-1161.92** | **0.61** |
| AT2G46630 |  | Unknown | **-16.73** | **0.6** |
| AT1G77120 | ADH | alcohol dehydrogenase 1 | **-1957.5** | **0.55** |
| AT3G03270 |  | Adenine nucleotide alpha hydrolases-like superfamily protein | **-490.84** | **0.51** |
| AT2G17850 |  | Rhodanese/Cell cycle control phosphatase superfamily protein | **-279.68** | **0.41** |
| *Group 2: : Post-transcriptionally regulated genes* | | | | |
| AT1G14370 | APK2A | protein kinase 2A | 3.42 | **6.83** |
| AT1G31870 |  | Unknown | -0.10 | **4.23** |
| AT2G37520 |  | Acyl-CoA N-acyltransferase with RING/FYVE/PHD-type zinc finger domain | | **3.19** |
| AT3G50470 | HR3 | homolog of RPW8 3 | | **3.08** |
| AT1G61470 |  | Polynucleotidyl transferase, ribonuclease H-like superfamily protein | 2.72 | **3.01** |
| AT5G49580 |  | Chaperone DnaJ-domain superfamily protein | | **2.4** |
| AT1G03850 | GRXS13 | Glutaredoxin family protein | 9.16 | **2.35** |
| AT1G26560 | BGLU40 | beta glucosidase 40 | | **2.15** |
| AT5G22450 |  | Unknown |  | **2.12** |
| AT3G25820 | TPS-CIN | terpene synthase-like sequence-1,8-cineole | 5.64 | **2.11** |
| AT4G08250 |  | GRAS family transcription factor | | **2.06** |
| AT5G17440 |  | LUC7 related protein | | **2.05** |
| AT2G22790 |  | Unknown |  | **2.04** |
| AT5G64200 | SC35 | ortholog of human splicing factor SC35 | | **1.99** |
| AT1G51820 |  | Leucine-rich repeat protein kinase family protein | | **1.97** |
| AT3G55280 | RPL23A2 | ribosomal protein L23AB | | **1.96** |
| AT1G78320 | GSTU23 | glutathione S-transferase TAU 23 | | **1.88** |
| AT1G19770 | PUP14 | purine permease 14 | 63.08 | **1.85** |
| AT3G16010 |  | Pentatricopeptide repeat (PPR-like) superfamily protein | | **1.83** |
| AT4G34135 | UGT73B2 | UDP-glucosyltransferase 73B2 | | **1.83** |
| AT5G10870 | CM2 | chorismate mutase 2 | | **1.83** |
| AT1G25520 |  | Uncharacterized protein family (UPF0016) | 6.53 | **1.82** |
| AT3G29410 |  | Terpenoid cyclases/Protein prenyltransferases superfamily protein | | **1.8** |
| AT1G74120 |  | Mitochondrial transcription termination factor family protein | | **1.79** |
| AT3G03340 | UNE6 | LUC7 related protein | | **1.79** |
| AT5G14740 | BETA CA2 | carbonic anhydrase 2 | | **1.77** |
| AT1G78370 | GSTU20 | glutathione S-transferase TAU 20 | | **1.76** |
| AT3G29400 | EXO70E1 | exocyst subunit exo70 family protein E1 | | **1.76** |
| AT3G47210 |  | Plant protein of unknown function (DUF247) | | **1.75** |
| AT2G29210 |  | splicing factor PWI domain-containing protein | | **1.74** |
| AT3G23810 | SAHH2 | S-adenosyl-l-homocysteine (SAH) hydrolase 2 | | **1.73** |
| AT1G02920 | GST11 | glutathione S-transferase 7 | -3.68 | **1.7** |
| AT5G20240 | PI | K-box region and MADS-box transcription factor family protein | | **1.7** |
| AT5G40280 | FTB | Prenyltransferase family protein | | **1.7** |
| AT1G29820 |  | Magnesium transporter CorA-like family protein | 0.61 | **1.69** |
| AT1G78340 | GSTU22 | glutathione S-transferase TAU 22 | | **1.69** |
| AT4G13940 | SAHH1 | S-adenosyl-L-homocysteine hydrolase | | **1.69** |
| AT2G21410 | VHA-A2 | vacuolar proton ATPase A2 | | **1.68** |
| AT3G16400 | MLP-470 | nitrile specifier protein 1 | | **1.68** |
| AT2G01520 | MLP328 | MLP-like protein 328 | | **1.67** |
| AT4G19180 |  | GDA1/CD39 nucleoside phosphatase family protein | | **1.67** |
| AT4G31880 |  | Unknown |  | **1.67** |
| AT5G16970 | AER | alkenal reductase | | **1.66** |
| AT5G23130 |  | Peptidoglycan-binding LysM domain-containing protein | | **1.66** |
| AT2G17525 |  | Pentatricopeptide repeat (PPR) superfamily protein | | **1.65** |
| AT1G17180 | GSTU25 | glutathione S-transferase TAU 25 | 22.59 | **1.64** |
| AT2G18690 |  | Unknown |  | **1.64** |
| AT2G38760 | ANN3 | annexin 3 |  | **1.62** |
| AT2G43820 | SAGT1 | UDP-glucosyltransferase 74F2 | | **1.62** |
| AT3G01420 | ALPHA-DOX1 | Peroxidase superfamily protein | | **1.62** |
| AT4G15550 | IAGLU | indole-3-acetate beta-D-glucosyltransferase | | **1.62** |
| AT5G41770 |  | crooked neck protein, putative / cell cycle protein, putative | | **1.62** |
| AT1G32640 | MYC2 | Basic helix-loop-helix (bHLH) DNA-binding family protein | 66.72 | **1.61** |
| AT1G68530 | CER6 | 3-ketoacyl-CoA synthase 6 | | **1.61** |
| AT4G00238 |  | DNA-binding storekeeper protein-related transcriptional regulator | | **1.61** |
| AT4G34640 | ERG9 | squalene synthase 1 | | **1.61** |
| AT3G01160 |  | Unknown |  | **1.6** |
| AT3G48090 | EDS1 | alpha/beta-Hydrolases superfamily protein | | **1.6** |
| AT3G57260 | PR2 | beta-1,3-glucanase 2 | | **1.6** |
| AT1G80930 |  | MIF4G domain-containing protein / MA3 domain-containing protein | | **1.59** |
| AT4G34138 | UGT73B1 | UDP-glucosyl transferase 73B1 | | **1.59** |
| AT5G16010 |  | 3-oxo-5-alpha-steroid 4-dehydrogenase family protein | | **1.59** |
| ATMG00090 |  | structural constituent of ribosome;protein binding | | **1.59** |
| AT4G20360 | RAB8D | RAB GTPase homolog E1B | | **1.58** |
| AT4G37640 | ACA2 | calcium ATPase 2 | | **1.58** |
| AT5G43300 | GDPD3 | PLC-like phosphodiesterases superfamily protein | | **1.58** |
| AT5G47880 | ERF1-1 | eukaryotic release factor 1-1 | | **1.58** |
| AT1G18840 | IQD30 | IQ-domain 30 | -1.49 | **1.57** |
| AT1G57720 |  | Translation elongation factor EF1B, gamma chain | 9.38 | **1.57** |
| AT1G72290 |  | Kunitz family trypsin and protease inhibitor protein | | **1.57** |
| AT5G13370 |  | Auxin-responsive GH3 family protein | | **1.57** |
| AT5G23405 |  | HMG-box (high mobility group) DNA-binding family protein | | **1.57** |
| AT1G17170 | GSTU24 | glutathione S-transferase TAU 24 | 24.51 | **1.56** |
| AT3G46790 | CRR2 | Tetratricopeptide repeat (TPR)-like superfamily protein | | **1.56** |
| AT4G17190 | FPS2 | farnesyl diphosphate synthase 2 | | **1.56** |
| AT4G32620 |  | Enhancer of polycomb-like transcription factor protein | | **1.56** |
| AT5G26000 | TGG1 | thioglucoside glucohydrolase 1 | | **1.56** |
| AT2G40650 |  | PRP38 family protein | | **1.55** |
| AT3G46690 |  | UDP-Glycosyltransferase superfamily protein | | **1.55** |
| AT5G28840 | GME | GDP-D-mannose 3',5'-epimerase | | **1.55** |
| AT2G02560 | CAND1 | cullin-associated and neddylation dissociated | | **1.54** |
| AT1G23060 |  | Unknown |  | **1.53** |
| AT3G23370 |  | RNA-binding (RRM/RBD/RNP motifs) family protein | | **1.53** |
| AT4G23100 | ECS1 | glutamate-cysteine ligase | | **1.53** |
| AT4G23510 |  | Disease resistance protein (TIR-NBS-LRR class) family | | **1.53** |
| AT1G03210 |  | Phenazine biosynthesis PhzC/PhzF protein | 3.28 | **1.52** |
| AT1G16400 | CYP79F2 | cytochrome P450, family 79, subfamily F, polypeptide 2 | 19.99 | **1.52** |
| AT2G45030 |  | Translation elongation factor EFG/EF2 protein | | **1.52** |
| AT3G57150 | CBF5 | homologue of NAP57 | | **1.52** |
| AT4G35160 |  | O-methyltransferase family protein | | **1.52** |
| AT5G36160 |  | Tyrosine transaminase family protein | | **1.52** |
| AT1G18740 |  | Protein of unknown function (DUF793) | 44.22 | **1.51** |
| AT1G33220 |  | Glycosyl hydrolase superfamily protein | | **1.51** |
| AT1G70850 | MLP34 | MLP-like protein 34 | | **1.51** |
| AT3G58110 |  | Unknown |  | **1.51** |
| AT5G27470 |  | seryl-tRNA synthetase / serine--tRNA ligase | | **1.51** |
| AT5G42820 | U2AF35B | Zinc finger C-x8-C-x5-C-x3-H type family protein | | **1.51** |
| AT2G21600 | RER1B | endoplasmatic reticulum retrieval protein 1B | | **1.5** |
| AT3G58610 |  | ketol-acid reductoisomerase | | **1.5** |
| AT5G36300 |  | Tetratricopeptide repeat (TPR)-like superfamily protein | | **1.5** |
| AT5G47100 | CBL9 | calcineurin B-like protein 9 | | **1.5** |
| AT1G12570 |  | Glucose-methanol-choline (GMC) oxidoreductase family protein | | **1.49** |
| AT1G43140 |  | Cullin family protein | | **1.49** |
| AT1G33780 |  | Protein of unknown function (DUF179) | -0.19 | **1.48** |
| AT2G26830 | emb1187 | Protein kinase superfamily protein | | **1.48** |
| AT2G28520 | VHA-A1 | vacuolar proton ATPase A1 | | **1.48** |
| AT2G44760 |  | Domain of unknown function (DUF3598) | | **1.48** |
| AT3G23670 | KINESIN-12B | phragmoplast-associated kinesin-related protein, putative | | **1.48** |
| AT4G13550 |  | triglyceride lipases;triglyceride lipases | | **1.48** |
| AT4G34131 | UGT73B3 | UDP-glucosyl transferase 73B3 | | **1.48** |
| AT5G02500 | -1 | heat shock cognate protein 70-1 | | **1.48** |
| AT5G34850 | PAP26 | purple acid phosphatase 26 | | **1.48** |
| AT5G40760 | G6PD6 | glucose-6-phosphate dehydrogenase 6 | | **1.48** |
| AT1G02130 | ARA-5 | RAS 5 | 9.61 | **1.47** |
| AT1G50380 |  | Prolyl oligopeptidase family protein | -0.54 | **1.47** |
| AT1G78160 | APUM7 | pumilio 7 |  | **1.47** |
| AT2G36830 | GAMMA-TIP | gamma tonoplast intrinsic protein | | **1.47** |
| AT3G03190 | GSTF11 | glutathione S-transferase F11 | | **1.47** |
| AT3G13330 | PA200 | proteasome activating protein 200 | | **1.47** |
| AT3G19760 | EIF4A-III | eukaryotic initiation factor 4A-III | | **1.47** |
| AT4G02420 |  | Concanavalin A-like lectin protein kinase family protein | | **1.47** |
| AT4G10280 |  | RmlC-like cupins superfamily protein | | **1.47** |
| AT4G12790 |  | P-loop containing nucleoside triphosphate hydrolases superfamily protein | | **1.47** |
| AT5G37760 |  | Chaperone DnaJ-domain superfamily protein | | **1.47** |
| AT5G42830 |  | HXXXD-type acyl-transferase family protein | | **1.47** |
| AT1G17190 | GSTU26 | glutathione S-transferase tau 26 | 10.53 | **1.46** |
| AT1G70890 | MLP43 | MLP-like protein 43 | | **1.46** |
| AT1G72440 | EDA25 | CCAAT-binding factor | | **1.46** |
| AT2G16940 |  | Splicing factor, CC1-like | | **1.46** |
| AT2G16950 | TRN1 | transportin 1 |  | **1.46** |
| AT2G44620 | MTACP-1 | mitochondrial acyl carrier protein 1 | | **1.46** |
| AT4G16570 | PRMT7 | protein arginine methyltransferase 7 | | **1.46** |
| AT5G19860 |  | Protein of unknown function, DUF538 | | **1.46** |
| AT3G53420 | PIP2 | plasma membrane intrinsic protein 2A | | **1.45** |
| AT4G30310 |  | FGGY family of carbohydrate kinase | | **1.45** |
| AT4G37270 | HMA1 | heavy metal atpase 1 | | **1.45** |
| AT5G05890 |  | UDP-Glycosyltransferase superfamily protein | | **1.45** |
| AT5G13520 |  | peptidase M1 family protein | | **1.45** |
| AT5G18410 | SRA1 | transcription activators | | **1.45** |
| AT5G23840 |  | MD-2-related lipid recognition domain-containing protein | | **1.45** |
| AT5G44120 | CRA1 | RmlC-like cupins superfamily protein | | **1.45** |
| AT5G50680 | SAE1B | SUMO activating enzyme 1B | | **1.45** |
| AT1G56590 | ZIP4 | Clathrin adaptor complexes medium subunit family protein | 0.87 | **1.44** |
| AT2G24300 |  | Calmodulin-binding protein | | **1.44** |
| AT2G37170 | PIP2;2 | plasma membrane intrinsic protein 2 | | **1.44** |
| AT3G21820 | XR2 | histone-lysine N-methyltransferase ATXR2 | | **1.44** |
| AT3G55760 |  | Unknown |  | **1.44** |
| AT5G27120 |  | NOP56-like pre RNA processing ribonucleoprotein | | **1.44** |
| ATCG00500 | ACCD | acetyl-CoA carboxylase carboxyl transferase subunit beta | | **1.44** |
| AT1G03365 |  | RING/U-box superfamily protein | -0.18 | **1.43** |
| AT1G61730 |  | DNA-binding storekeeper protein-related transcriptional regulator | 1.15 | **1.43** |
| AT2G30620 |  | winged-helix DNA-binding transcription factor family protein | | **1.43** |
| AT3G14680 | CYP72A14 | cytochrome P450, family 72, subfamily A, polypeptide 14 | | **1.43** |
| AT3G30390 |  | Transmembrane amino acid transporter family protein | | **1.43** |
| AT4G25260 |  | Plant invertase/pectin methylesterase inhibitor superfamily protein | | **1.43** |
| AT4G33390 |  | Plant protein of unknown function (DUF827) | | **1.43** |
| AT4G33565 |  | RING/U-box superfamily protein | | **1.43** |
| AT5G43400 |  | Uncharacterised conserved protein UCP015417, vWA | | **1.43** |
| AT1G05680 | UGT74E2 | Uridine diphosphate glycosyltransferase 74E2 | 4.89 | **1.42** |
| AT1G18700 |  | DNAJ heat shock N-terminal domain-containing protein | -0.20 | **1.42** |
| AT1G54270 | EIF4A-2 | eif4a-2 | 3.35 | **1.42** |
| AT1G51840 |  | protein kinase-related | | **1.42** |
| AT1G65930 | cICDH | cytosolic NADP+-dependent isocitrate dehydrogenase | | **1.42** |
| AT1G69670 | CUL3B | cullin 3B |  | **1.42** |
| AT1G73260 | KTI1 | kunitz trypsin inhibitor 1 | | **1.42** |
| AT2G34020 |  | Calcium-binding EF-hand family protein | | **1.42** |
| AT2G42370 |  | Unknown |  | **1.42** |
| AT3G09440 |  | Heat shock protein 70 (Hsp 70) family protein | | **1.42** |
| AT4G22340 | CDS2 | cytidinediphosphate diacylglycerol synthase 2 | | **1.42** |
| AT4G33090 | APM1 | aminopeptidase M1 | | **1.42** |
| AT5G12420 |  | O-acyltransferase (WSD1-like) family protein | | **1.42** |
| AT5G59420 | ORP3C | OSBP(oxysterol binding protein)-related protein 3C | | **1.42** |
| AT5G66930 |  | Unknown |  | **1.42** |
| AT1G06720 |  | P-loop containing nucleoside triphosphate hydrolases superfamily protein | -2.39 | **1.41** |
| AT1G60420 |  | DC1 domain-containing protein | 0.36 | **1.41** |
| AT1G01030 | NGA3 | AP2/B3-like transcriptional factor family protein | | **1.41** |
| AT1G73920 |  | alpha/beta-Hydrolases superfamily protein | | **1.41** |
| AT1G80700 |  | Unknown |  | **1.41** |
| AT2G03120 | SPP | signal peptide peptidase | | **1.41** |
| AT2G15695 |  | Protein of unknown function DUF829, transmembrane 53 | | **1.41** |
| AT2G29420 | GSTU7 | glutathione S-transferase tau 7 | | **1.41** |
| AT3G13720 | PRA1.F3 | PRA1 (Prenylated rab acceptor) family protein | | **1.41** |
| AT3G19990 |  | Unknown |  | **1.41** |
| AT4G36070 | CPK18 | calcium-dependent protein kinase 18 | | **1.41** |
| AT4G38240 | CGL | alpha-1,3-mannosyl-glycoprotein beta-1,2-N-acetylglucosaminyltransferase, putative | | **1.41** |
| AT5G03520 | RAB-E1D | RAB GTPase homolog 8C | | **1.41** |
| AT5G23920 |  | Unknown |  | **1.41** |
| AT5G45490 |  | P-loop containing nucleoside triphosphate hydrolases superfamily protein | | **1.41** |
| AT1G17860 |  | Kunitz family trypsin and protease inhibitor protein | 40.72 | **1.4** |
| AT1G51060 | HTA10 | histone H2A 10 | 18.83 | **1.4** |
| AT1G73740 |  | UDP-Glycosyltransferase superfamily protein | | **1.4** |
| AT3G53780 | RBL4 | RHOMBOID-like protein 4 | | **1.4** |
| AT5G18570 | OBGC | GTP1/OBG family protein | | **1.4** |
| AT1G52200 |  | PLAC8 family protein | 33.12 | **1.39** |
| AT1G63770 |  | Peptidase M1 family protein | -0.91 | **1.39** |
| AT2G37970 | SOUL-1 | SOUL heme-binding family protein | | **1.39** |
| AT2G40300 | FER4 | ferritin 4 |  | **1.39** |
| AT3G10260 |  | Reticulon family protein | | **1.39** |
| AT4G03560 | CCH1 | two-pore channel 1 | | **1.39** |
| AT4G13050 |  | Acyl-ACP thioesterase | | **1.39** |
| AT4G32250 |  | Protein kinase superfamily protein | | **1.39** |
| AT4G33150 | LKR | lysine-ketoglutarate reductase/saccharopine dehydrogenase bifunctional enzyme | | **1.39** |
| AT5G18640 |  | alpha/beta-Hydrolases superfamily protein | | **1.39** |
| AT5G62480 | GSTU9 | glutathione S-transferase tau 9 | | **1.39** |
| AT2G32930 | ZFN2 | zinc finger nuclease 2 | | **0.75** |
| AT4G17550 | G3Pp4 | Major facilitator superfamily protein | | **0.75** |
| AT4G29910 | ORC5 | origin recognition complex protein 5 | | **0.75** |
| AT5G21280 |  | hydroxyproline-rich glycoprotein family protein | | **0.75** |
| AT1G03120 | RAB28 | responsive to abscisic acid 28 | | **0.74** |
| AT1G24020 | MLP423 | MLP-like protein 423 | | **0.74** |
| AT4G12580 |  | Unknown |  | **0.74** |
| AT4G37970 | CAD6 | cinnamyl alcohol dehydrogenase 6 | | **0.74** |
| AT4G39170 |  | Sec14p-like phosphatidylinositol transfer family protein | | **0.74** |
| AT5G01870 |  | Bifunctional inhibitor/lipid-transfer protein/seed storage 2S albumin superfamily protein | | **0.74** |
| AT5G48450 | sks3 | SKU5 similar 3 | | **0.74** |
| AT5G52200 | I-2 | phosphoprotein phosphatase inhibitors | | **0.74** |
| AT1G09140 | SR30 | SERINE-ARGININE PROTEIN 30 | 23.29 | **0.73** |
| AT1G10990 |  | Unknown | -5.59 | **0.73** |
| AT1G15280 |  | CASC3/Barentsz eIF4AIII binding | 5.21 | **0.73** |
| AT1G63640 |  | P-loop nucleoside triphosphate hydrolases superfamily protein with CH (Calponin Homology) domain | -1.19 | **0.73** |
| AT2G42870 | HLH1 | phy rapidly regulated 1 | | **0.73** |
| AT3G16920 | CTL2 | chitinase-like protein 2 | | **0.73** |
| AT3G19780 |  | Unknown |  | **0.73** |
| AT3G23470 |  | Cyclopropane-fatty-acyl-phospholipid synthase | | **0.73** |
| AT1G04990 |  | Zinc finger C-x8-C-x5-C-x3-H type family protein | -1.67 | **0.72** |
| AT1G57943 | PUP17 | purine permease 17 | | **0.72** |
| AT1G61215 | BRD4 | bromodomain 4 | | **0.72** |
| AT1G76120 |  | Pseudouridine synthase family protein | | **0.72** |
| AT2G15660 | AGL95 | AGAMOUS-like 95 | | **0.72** |
| AT3G12900 |  | 2-oxoglutarate (2OG) and Fe(II)-dependent oxygenase superfamily protein | | **0.72** |
| AT3G17680 |  | Kinase interacting (KIP1-like) family protein | | **0.72** |
| AT3G45070 |  | P-loop containing nucleoside triphosphate hydrolases superfamily protein | | **0.72** |
| AT4G38560 |  | Arabidopsis phospholipase-like protein (PEARLI 4) family | | **0.72** |
| AT5G59910 | HTB4 | Histone superfamily protein | | **0.72** |
| AT1G48750 |  | Bifunctional inhibitor/lipid-transfer protein/seed storage 2S albumin superfamily protein | | **0.71** |
| AT1G52450 |  | Ubiquitin carboxyl-terminal hydrolase-related protein | | **0.71** |
| AT1G80640 |  | Protein kinase superfamily protein | | **0.71** |
| AT3G17160 |  | Unknown |  | **0.71** |
| AT3G18900 |  | Unknown |  | **0.71** |
| AT3G49055 |  | Unknown |  | **0.71** |
| AT1G20440 | COR47 | cold-regulated 47 | 128.63 | **0.7** |
| AT2G45070 | SEC61 BETA | Preprotein translocase Sec, Sec61-beta subunit protein | | **0.7** |
| AT3G07660 |  | Kinase-related protein of unknown function (DUF1296) | | **0.7** |
| AT3G13570 | SCL30A | SC35-like splicing factor 30A | | **0.7** |
| AT3G19210 | RAD54 | homolog of RAD54 | | **0.7** |
| AT5G13350 |  | Auxin-responsive GH3 family protein | | **0.7** |
| AT1G62150 |  | Mitochondrial transcription termination factor family protein | -0.35 | **0.69** |
| AT1G64880 |  | Ribosomal protein S5 family protein | -0.44 | **0.69** |
| AT2G28490 |  | RmlC-like cupins superfamily protein | | **0.69** |
| AT3G55950 | CRR3 | CRINKLY4 related 3 | | **0.69** |
| AT2G18328 | RL4 | RAD-like 4 |  | **0.68** |
| AT2G45280 | RAD51C | RAS associated with diabetes protein 51C | | **0.68** |
| AT3G48610 | NPC6 | non-specific phospholipase C6 | | **0.68** |
| AT5G22650 | HD2 | histone deacetylase 2B | | **0.68** |
| AT5G46390 |  | Peptidase S41 family protein | | **0.68** |
| AT5G47820 | FRA1 | P-loop containing nucleoside triphosphate hydrolases superfamily protein | | **0.68** |
| AT1G52690 | LEA7 | Late embryogenesis abundant protein (LEA) family protein | -9.11 | **0.67** |
| AT1G66980 | GDPDL2 | suppressor of npr1-1 constitutive 4 | | **0.67** |
| AT1G69770 | CMT3 | chromomethylase 3 | | **0.67** |
| AT2G26040 | PYL2 | PYR1-like 2 |  | **0.67** |
| AT2G41445 |  | Unknown |  | **0.67** |
| AT3G45730 |  | Unknown |  | **0.67** |
| AT3G53210 |  | nodulin MtN21 /EamA-like transporter family protein | | **0.67** |
| AT4G26780 | AR192 | Co-chaperone GrpE family protein | | **0.67** |
| AT5G08050 |  | Protein of unknown function (DUF1118) | | **0.66** |
| AT4G28090 | sks10 | SKU5 similar 10 | | **0.65** |
| AT5G67385 |  | Phototropic-responsive NPH3 family protein | | **0.65** |
| AT1G65480 | FT | PEBP (phosphatidylethanolamine-binding protein) family protein | | **0.64** |
| AT4G30930 | NFD1 | Ribosomal protein L21 | | **0.64** |
| AT4G37080 |  | Protein of unknown function, DUF547 | | **0.64** |
| AT5G47550 |  | Cystatin/monellin superfamily protein | | **0.64** |
| AT1G55370 | NDF5 | NDH-dependent cyclic electron flow 5 | | **0.63** |
| AT5G62550 |  | Unknown |  | **0.63** |
| AT3G15353 | MT3 | metallothionein 3 | | **0.62** |
| AT3G55460 | SCL30 | SC35-like splicing factor 30 | | **0.62** |
| AT2G43200 |  | S-adenosyl-L-methionine-dependent methyltransferases superfamily protein | | **0.61** |
| AT2G45406 |  | Galactose oxidase/kelch repeat superfamily protein | | **0.61** |
| AT5G60270 |  | Concanavalin A-like lectin protein kinase family protein | 2.24 | **0.6** |
| AT4G32510 |  | HCO3- transporter family | | **0.6** |
| AT3G63300 | FKD1 | FORKED 1 |  | **0.59** |
| AT4G14330 |  | P-loop containing nucleoside triphosphate hydrolases superfamily protein | | **0.59** |
| AT2G24960 |  | Unknown |  | **0.58** |
| AT3G60310 |  | Unknown |  | **0.58** |
| AT4G29880 | PIRL7 | plant intracellular ras group-related LRR 7 | | **0.58** |
| AT5G19151 |  | Unknown |  | **0.58** |
| AT5G55020 | MYB120 | myb domain protein 120 | | **0.58** |
| AT2G20440 |  | Ypt/Rab-GAP domain of gyp1p superfamily protein | | **0.56** |
| AT4G26740 | PXG1 | seed gene 1 |  | **0.53** |
| AT1G28290 | AGP31 | arabinogalactan protein 31 | -206.72 | **0.52** |
| ATCG00710 | PSBH | photosystem II reaction center protein H | | **0.5** |
| *Group 3: Solely transcriptionally regulated genes* | | | | |
| AT3G09260 | BGLU23 | Glycosyl hydrolase superfamily protein | **935.1** | 1.13 |
| AT2G33830 |  | Dormancy/auxin associated family protein | **498.59** | 1.39 |
| AT3G16420 | JAL30 | PYK10-binding protein 1 | **321.51** | 1.07 |
| AT3G61430 | PIP1 | plasma membrane intrinsic protein 1A | **259.59** | 1.43 |
| AT2G22122 |  | Unknown | **189.6** | 1.14 |
| AT1G78380 | GSTU19 | glutathione S-transferase TAU 19 | **177.81** | 1.24 |
| AT1G07590 |  | Tetratricopeptide repeat (TPR)-like superfamily protein | **174.08** | 0.82 |
| AT4G16190 |  | Papain family cysteine protease | **173.85** | 1.25 |
| AT2G47730 | GSTF5 | glutathione S-transferase phi 8 | **162.83** | 0.93 |
| AT1G66270 | BGLU21 | Glycosyl hydrolase superfamily protein | **160.72** | 1.22 |
| AT1G75750 | GASA1 | GAST1 protein homolog 1 | **155.6** | 1.07 |
| AT5G11670 | NADP-ME2 | NADP-malic enzyme 2 | **153.79** | 1.12 |
| AT3G48360 | BT2 | BTB and TAZ domain protein 2 | **152.29** |  |
| AT2G30870 | GSTF10 | glutathione S-transferase PHI 10 | **141.57** | 1.31 |
| AT4G38080 |  | hydroxyproline-rich glycoprotein family protein | **135.08** |  |
| AT5G63160 | BT1 | BTB and TAZ domain protein 1 | **125.62** |  |
| AT3G18780 | ACT2 | actin 2 | **116.94** |  |
| AT4G17340 | DELTA-TIP2 | tonoplast intrinsic protein 2;2 | **116.18** | 1.35 |
| AT4G21850 | MSRB9 | methionine sulfoxide reductase B9 | **115.96** | 1.02 |
| AT1G08830 | CSD1 | copper/zinc superoxide dismutase 1 | **114.91** | 1.02 |
| AT3G10985 | WI-12 | senescence associated gene 20 | **114.25** |  |
| AT4G30170 |  | Peroxidase family protein | **112.17** | 1.03 |
| AT1G56220 |  | Dormancy/auxin associated family protein | **105.77** | 0.94 |
| AT4G20260 | PCAP1 | plasma-membrane associated cation-binding protein 1 | **104.49** | 1.22 |
| AT1G11185 |  | other RNA | **103.87** |  |
| AT5G23020 | IMS2 | 2-isopropylmalate synthase 2 | **102.33** | 1.39 |
| AT4G23400 | PIP1;5 | plasma membrane intrinsic protein 1;5 | **102.16** | 1.29 |
| AT3G25830 | TPS-CIN | terpene synthase-like sequence-1,8-cineole | **97.45** |  |
| AT4G29905 |  | Unknown | **94.06** |  |
| AT1G67870 |  | glycine-rich protein | **93.79** |  |
| AT3G49780 | PSK3 (FORMER SYMBOL) | phytosulfokine 4 precursor | **87.8** | 0.92 |
| AT1G77330 |  | 2-oxoglutarate (2OG) and Fe(II)-dependent oxygenase superfamily protein | **86.25** | 0.92 |
| AT1G49240 | ACT8 | actin 8 | **85.37** | 1.11 |
| AT1G14870 | PCR2 | PLANT CADMIUM RESISTANCE 2 | **84.09** | 1.19 |
| AT3G15950 | NAI2 | DNA topoisomerase-related | **81.69** | 1.20 |
| AT4G01450 |  | nodulin MtN21 /EamA-like transporter family protein | **81.41** |  |
| AT3G21510 | AHP1 | histidine-containing phosphotransmitter 1 | **77.97** | 0.95 |
| AT1G54000 | GLL22 | GDSL-like Lipase/Acylhydrolase superfamily protein | **77.94** | 1.09 |
| AT1G76680 | OPR1 | 12-oxophytodienoate reductase 1 | **77.7** | 1.19 |
| AT5G04340 | C2H2 | zinc finger of Arabidopsis thaliana 6 | **76.54** |  |
| AT5G17330 | GAD | glutamate decarboxylase | **76.22** | 1.26 |
| AT5G08790 | anac081 | NAC (No Apical Meristem) domain transcriptional regulator superfamily protein | **76.02** |  |
| AT5G56540 | AGP14 | arabinogalactan protein 14 | **73.54** |  |
| AT4G34050 | CCoAOMT1 | S-adenosyl-L-methionine-dependent methyltransferases superfamily protein | **72.97** | 0.88 |
| AT1G35580 | A/N-InvG | cytosolic invertase 1 | **72.75** | 1.23 |
| AT4G17500 | ERF-1 | ethylene responsive element binding factor 1 | **72.74** |  |
| AT3G48990 |  | AMP-dependent synthetase and ligase family protein | **72.69** | 1.20 |
| AT4G33420 |  | Peroxidase superfamily protein | **72.52** | 1.06 |
| AT4G04830 | MSRB5 | methionine sulfoxide reductase B5 | **72.38** | 1.01 |
| AT4G40040 |  | Histone superfamily protein | **72.19** |  |
| AT3G50970 | LTI30 | dehydrin family protein | **71.95** | 1.21 |
| AT4G35100 | PIP2;7 | plasma membrane intrinsic protein 3 | **68.33** | 1.24 |
| AT2G43910 | HOL1 | HARMLESS TO OZONE LAYER 1 | **68** | 1.28 |
| AT1G21100 | IGMT1 | O-methyltransferase family protein | **67.27** | 1.27 |
| AT5G59090 | SBT4.12 | subtilase 4.12 | **66.6** | 1.36 |
| AT5G53250 | AGP22 | arabinogalactan protein 22 | **66.28** |  |
| AT2G32150 |  | Haloacid dehalogenase-like hydrolase (HAD) superfamily protein | **66.07** | 0.97 |
| AT3G48340 | CEP2 | Cysteine proteinases superfamily protein | **64.36** | 1.24 |
| AT1G09970 | LRR XI-23 | Leucine-rich receptor-like protein kinase family protein | **63.8** | 1.24 |
| AT5G09530 | PELPK1 | hydroxyproline-rich glycoprotein family protein | **63.45** | 1.16 |
| AT4G31500 | R4 | cytochrome P450, family 83, subfamily B, polypeptide 1 | **62.13** | 1.34 |
| AT3G51910 | -HSFA7A | heat shock transcription factor A7A | **59.82** |  |
| AT4G13180 |  | NAD(P)-binding Rossmann-fold superfamily protein | **54.24** | 1.07 |
| AT3G11280 |  | Duplicated homeodomain-like superfamily protein | **53.99** |  |
| AT5G63790 | ANAC102 | NAC domain containing protein 102 | **53.02** |  |
| AT1G80380 |  | P-loop containing nucleoside triphosphate hydrolases superfamily protein | **52.73** | 1.04 |
| AT5G54500 | FQR1 | flavodoxin-like quinone reductase 1 | **52.73** | 0.99 |
| AT4G35090 | CAT2 | catalase 2 | **52.69** | 1.23 |
| AT3G06390 |  | Uncharacterised protein family (UPF0497) | **50.48** | 1.41 |
| AT5G56870 | BGAL4 | beta-galactosidase 4 | **50.09** | 1.17 |
| AT5G63660 | LCR74 | Scorpion toxin-like knottin superfamily protein | **50.01** |  |
| AT5G35190 | EXT13 | proline-rich extensin-like family protein | **48.62** |  |
| AT5G03630 | MDAR2 | Pyridine nucleotide-disulphide oxidoreductase family protein | **48.57** | 1.07 |
| AT2G46600 |  | Calcium-binding EF-hand family protein | **48.45** |  |
| AT3G53990 |  | Adenine nucleotide alpha hydrolases-like superfamily protein | **47.39** | 0.99 |
| AT5G10430 | AGP4 | arabinogalactan protein 4 | **47.16** |  |
| AT1G64230 | UBC28 | ubiquitin-conjugating enzyme 28 | **47.09** | 0.91 |
| AT5G23010 | IMS3 | methylthioalkylmalate synthase 1 | **46.67** | 1.32 |
| AT3G61260 |  | Remorin family protein | **45.77** | 1.04 |
| AT5G66170 | STR18 | sulfurtransferase 18 | **45.03** | 1.09 |
| AT5G64400 |  | Unknown | **44.35** | 0.76 |
| AT4G14130 | XTH15 | xyloglucan endotransglucosylase/hydrolase 15 | **44.07** | 1.12 |
| AT3G01290 | HIR2 | SPFH/Band 7/PHB domain-containing membrane-associated protein family | **43.89** | 1.37 |
| AT4G01480 | PPa5 | pyrophosphorylase 5 | **43.72** | 1.04 |
| AT4G37010 | CEN2 | centrin 2 | **43.66** | 1.16 |
| AT5G19230 |  | Glycoprotein membrane precursor GPI-anchored | **42.99** | 1.29 |
| AT1G77885 |  | Unknown | **42.81** |  |
| AT4G14030 | SBP1 | selenium-binding protein 1 | **42.19** | 0.99 |
| AT1G03870 | FLA9 | FASCICLIN-like arabinoogalactan 9 | **41.68** | 1.07 |
| AT2G17500 |  | Auxin efflux carrier family protein | **41.68** |  |
| AT5G49440 |  | Unknown | **41.21** |  |
| AT1G61740 |  | Sulfite exporter TauE/SafE family protein | **41.11** |  |
| AT1G09210 | CRT1b | calreticulin 1b | **40.87** | 0.92 |
| AT4G25170 |  | Uncharacterised conserved protein (UCP012943) | **39.35** |  |
| AT1G14200 |  | RING/U-box superfamily protein | **39.3** |  |
| AT4G37300 | MEE59 | maternal effect embryo arrest 59 | **39.27** | 0.92 |
| AT1G56340 | CRT1a | calreticulin 1a | **37.85** | 0.82 |
| AT1G64200 | VHA-E3 | vacuolar H+-ATPase subunit E isoform 3 | **37.23** | 1.18 |
| AT2G29490 | GSTU1 | glutathione S-transferase TAU 1 | **37.14** | 1.34 |
| AT2G40080 | ELF4 | Protein of unknown function (DUF1313) | **36.94** | 0.96 |
| AT5G26260 |  | TRAF-like family protein | **36.24** | 1.18 |
| AT3G13790 | BFRUCT1 | Glycosyl hydrolases family 32 protein | **35.46** | 1.07 |
| AT4G13390 | EXT12 | Proline-rich extensin-like family protein | **35.12** | 0.75 |
| AT1G78000 | SEL1 | sulfate transporter 1;2 | **34.83** | 1.13 |
| AT4G11600 | GPX6 | glutathione peroxidase 6 | **34.78** | 0.89 |
| AT4G23680 |  | Polyketide cyclase/dehydrase and lipid transport superfamily protein | **34.68** | 1.36 |
| AT5G19600 | SULTR3;5 | sulfate transporter 3;5 | **34.67** |  |
| AT1G62660 |  | Glycosyl hydrolases family 32 protein | **34.23** | 1.27 |
| AT5G47450 | TIP23 | tonoplast intrinsic protein 2;3 | **33.85** |  |
| AT1G21440 |  | Phosphoenolpyruvate carboxylase family protein | **33.73** | 1.23 |
| AT4G22666 |  | Bifunctional inhibitor/lipid-transfer protein/seed storage 2S albumin superfamily protein | **33.29** | 1.29 |
| AT2G19110 | HMA4 | heavy metal atpase 4 | **32.83** | 1.01 |
| AT4G32060 |  | calcium-binding EF hand family protein | **32.17** |  |
| AT1G23870 | TPS9 | trehalose-phosphatase/synthase 9 | **31.46** | 1.27 |
| AT2G40940 | ERS | ethylene response sensor 1 | **31.19** | 0.99 |
| AT4G33610 |  | glycine-rich protein | **31.13** |  |
| AT2G38400 | AGT3 | alanine:glyoxylate aminotransferase 3 | **31.05** | 1.03 |
| AT3G05950 |  | RmlC-like cupins superfamily protein | **31.01** | 1.08 |
| AT5G53560 | B5-A | cytochrome B5 isoform E | **30.99** | 0.87 |
| AT5G62340 |  | Plant invertase/pectin methylesterase inhibitor superfamily protein | **30.79** | 0.97 |
| AT2G43100 | LEUD1 | isopropylmalate isomerase 2 | **30.01** | 0.93 |
| AT2G22510 |  | hydroxyproline-rich glycoprotein family protein | **29.87** |  |
| AT2G36380 | ABCG34 | pleiotropic drug resistance 6 | **29.82** | 1.25 |
| AT5G13750 | ZIFL1 | zinc induced facilitator-like 1 | **29.66** |  |
| AT1G13300 | HRS1 | myb-like transcription factor family protein | **29.58** | 0.86 |
| AT2G28190 | CSD2 | copper/zinc superoxide dismutase 2 | **29.3** | 0.95 |
| AT1G08930 | ERD6 | Major facilitator superfamily protein | **29.24** | 1.17 |
| AT5G58110 |  | chaperone binding;ATPase activators | **28.97** | 0.98 |
| AT2G29450 | 103-1A | glutathione S-transferase tau 5 | **28.82** | 1.34 |
| AT3G17810 | PYD1 | pyrimidine 1 | **28.77** | 1.11 |
| AT1G72900 |  | Toll-Interleukin-Resistance (TIR) domain-containing protein | **28.76** |  |
| AT1G21750 | PDI5 | PDI-like 1-1 | **28.42** | 0.95 |
| AT1G30270 | CIPK23 | CBL-interacting protein kinase 23 | **28.31** | 0.99 |
| AT4G00880 |  | SAUR-like auxin-responsive protein family | **28.01** |  |
| AT5G02480 |  | HSP20-like chaperones superfamily protein | **27.79** |  |
| AT4G24190 | Hsp90-7 | Chaperone protein htpG family protein | **27.71** | 1.20 |
| AT5G48230 | ACAT2 | acetoacetyl-CoA thiolase 2 | **27.63** | 0.98 |
| AT3G01690 |  | alpha/beta-Hydrolases superfamily protein | **27.61** |  |
| AT2G02120 | LCR70 | Scorpion toxin-like knottin superfamily protein | **27.45** | 1.19 |
| AT2G20560 |  | DNAJ heat shock family protein | **27.42** | 1.04 |
| AT5G47200 | RAB1A | RAB GTPase homolog 1A | **27.4** | 1.18 |
| AT4G15990 |  | Unknown | **27.39** |  |
| AT1G76090 | SMT3 | sterol methyltransferase 3 | **26.99** | 1.32 |
| AT4G08300 |  | nodulin MtN21 /EamA-like transporter family protein | **26.84** |  |
| AT5G64240 | MC3 | metacaspase 3 | **26.73** | 1.18 |
| AT1G78660 | GGH1 | gamma-glutamyl hydrolase 1 | **26.72** | 1.13 |
| AT5G13080 | WRKY75 | WRKY DNA-binding protein 75 | **26.62** |  |
| AT2G18280 | TLP2 | tubby like protein 2 | **26.62** |  |
| AT3G45980 | H2B | Histone superfamily protein | **26.1** | 1.27 |
| AT1G56700 |  | Peptidase C15, pyroglutamyl peptidase I-like | **26.08** | 1.08 |
| AT4G36410 | UBC17 | ubiquitin-conjugating enzyme 17 | **25.64** |  |
| AT1G05010 | ACO4 | ethylene-forming enzyme | **25.63** | 1.21 |
| AT5G47770 | FPS1 | farnesyl diphosphate synthase 1 | **25.51** | 1.37 |
| AT3G53730 |  | Histone superfamily protein | **25.41** |  |
| AT2G01450 | MPK17 | MAP kinase 17 | **25.2** | 0.95 |
| AT4G17530 | RAB1C | RAB GTPase homolog 1C | **25.19** | 1.36 |
| AT3G19450 | CAD4 | GroES-like zinc-binding alcohol dehydrogenase family protein | **25.12** | 1.11 |
| AT1G78300 | 14-3-3OMEGA | general regulatory factor 2 | **24.79** | 1.05 |
| AT4G27320 | PHOS34 | Adenine nucleotide alpha hydrolases-like superfamily protein | **24.46** | 0.94 |
| AT1G54010 |  | GDSL-like Lipase/Acylhydrolase superfamily protein | **24.43** | 1.00 |
| AT3G27090 |  | DCD (Development and Cell Death) domain protein | **24.26** |  |
| AT5G10130 |  | Pollen Ole e 1 allergen and extensin family protein | **24.21** | 1.12 |
| AT4G34720 | VHA-C1 | ATPase, F0/V0 complex, subunit C protein | **24.13** |  |
| AT5G07322 |  | other RNA | **23.6** |  |
| AT2G40110 |  | Yippee family putative zinc-binding protein | **23.33** |  |
| AT2G19570 | CDA1 | cytidine deaminase 1 | **23.32** | 0.95 |
| AT4G24160 |  | alpha/beta-Hydrolases superfamily protein | **23.28** | 1.23 |
| AT2G47180 | GolS1 | galactinol synthase 1 | **23.11** |  |
| AT2G23030 | SNRK2-9 | SNF1-related protein kinase 2.9 | **23.03** |  |
| AT1G11450 |  | nodulin MtN21 /EamA-like transporter family protein | **22.68** |  |
| AT4G26080 | ABI1 | Protein phosphatase 2C family protein | **22.66** | 1.06 |
| AT2G22330 | CYP79B3 | cytochrome P450, family 79, subfamily B, polypeptide 3 | **22.41** |  |
| AT3G14067 |  | Subtilase family protein | **22.36** | 1.13 |
| AT5G59540 |  | 2-oxoglutarate (2OG) and Fe(II)-dependent oxygenase superfamily protein | **22.35** | 1.17 |
| AT5G15090 | VDAC3 | voltage dependent anion channel 3 | **22.32** | 0.92 |
| AT2G47160 | BOR1 | HCO3- transporter family | **22.17** |  |
| AT1G70410 | BCA4 | beta carbonic anhydrase 4 | **21.87** | 0.82 |
| AT1G64590 |  | NAD(P)-binding Rossmann-fold superfamily protein | **21.86** | 1.09 |
| AT4G00860 | 0ZI1 | Protein of unknown function (DUF1138) | **21.81** | 1.07 |
| AT5G57090 | AGR | Auxin efflux carrier family protein | **21.78** |  |
| AT3G54400 |  | Eukaryotic aspartyl protease family protein | **21.75** | 0.97 |
| AT4G19030 | 0 | NOD26-like major intrinsic protein 1 | **21.73** |  |
| AT1G73500 | MKK9 | MAP kinase kinase 9 | **21.56** |  |
| AT4G11360 | RHA1B | RING-H2 finger A1B | **21.49** |  |
| AT4G14410 | bHLH104 | basic helix-loop-helix (bHLH) DNA-binding superfamily protein | **21.46** |  |
| AT5G28050 |  | Cytidine/deoxycytidylate deaminase family protein | **21.43** | 0.88 |
| AT4G39940 | AKN2 | APS-kinase 2 | **21.31** | 1.13 |
| AT4G08290 |  | nodulin MtN21 /EamA-like transporter family protein | **21.24** |  |
| AT1G21000 |  | PLATZ transcription factor family protein | **21.19** |  |
| AT4G26220 |  | S-adenosyl-L-methionine-dependent methyltransferases superfamily protein | **20.97** | 1.12 |
| AT5G64130 |  | cAMP-regulated phosphoprotein 19-related protein | **20.8** | 0.83 |
| AT3G58680 | MBF1B | multiprotein bridging factor 1B | **20.76** | 1.02 |
| AT2G46750 |  | D-arabinono-1,4-lactone oxidase family protein | **20.71** | 1.38 |
| AT4G18340 |  | Glycosyl hydrolase superfamily protein | **20.61** |  |
| AT2G47270 | UPB1 | sequence-specific DNA binding transcription factors;transcription regulators | **20.61** |  |
| AT4G15760 | MO1 | monooxygenase 1 | **20.41** |  |
| AT1G18570 | MYB51 | myb domain protein 51 | **20.34** |  |
| AT4G06744 |  | Leucine-rich repeat (LRR) family protein | **20.3** | 1.09 |
| AT3G26470 |  | Powdery mildew resistance protein, RPW8 domain | **20.2** |  |
| AT2G30520 | RPT2 | Phototropic-responsive NPH3 family protein | **20.16** | 1.09 |
| AT2G01490 |  | phytanoyl-CoA dioxygenase (PhyH) family protein | **20.04** | 1.34 |
| AT4G33580 | BCA5 | beta carbonic anhydrase 5 | **19.99** | 1.18 |
| AT3G60130 | BGLU16 | beta glucosidase 16 | **19.95** | 1.26 |
| AT4G16760 | ACX1 | acyl-CoA oxidase 1 | **19.87** | 1.25 |
| AT1G76490 | HMGR1 | hydroxy methylglutaryl CoA reductase 1 | **19.86** | 0.99 |
| AT5G10830 |  | S-adenosyl-L-methionine-dependent methyltransferases superfamily protein | **19.74** | 1.02 |
| AT5G46790 | PYL1 | PYR1-like 1 | **19.63** | 1.04 |
| AT2G47000 | ABCB4 | ATP binding cassette subfamily B4 | **19.62** | 1.15 |
| AT1G12520 | CCS | copper chaperone for SOD1 | **19.61** | 0.96 |
| AT1G53910 | RAP2.12 | related to AP2 12 | **19.55** | 1.26 |
| AT5G23820 |  | MD-2-related lipid recognition domain-containing protein | **19.54** | 1.26 |
| AT4G36988 | CPuORF49 | conserved peptide upstream open reading frame 49 | **19.5** |  |
| AT4G36990 | -HSFB1 | heat shock factor 4 | **19.5** |  |
| AT5G18860 | NSH3 | inosine-uridine preferring nucleoside hydrolase family protein | **19.48** | 1.09 |
| AT1G64660 | MGL | methionine gamma-lyase | **19.44** | 1.16 |
| AT2G47130 | SDR3 | NAD(P)-binding Rossmann-fold superfamily protein | **19.41** | 1.09 |
| AT4G12390 | PME1 | pectin methylesterase inhibitor 1 | **19.3** | 1.08 |
| AT3G21520 | DMP1 | DUF679 domain membrane protein 1 | **19.18** | 1.28 |
| AT1G20260 |  | ATPase, V1 complex, subunit B protein | **19.03** | 1.18 |
| AT1G22360 | UGT85A2 | UDP-glucosyl transferase 85A2 | **19.02** | 1.32 |
| AT1G68440 |  | Unknown | **18.99** | 0.93 |
| AT2G44350 | CS | Citrate synthase family protein | **18.94** | 1.04 |
| AT3G25585 | AAPT2 | aminoalcoholphosphotransferase | **18.82** |  |
| AT1G34370 | STOP1 | C2H2 and C2HC zinc fingers superfamily protein | **18.68** |  |
| AT1G26800 |  | RING/U-box superfamily protein | **18.64** |  |
| AT3G04090 | SIP1;1 | small and basic intrinsic protein 1A | **18.48** | 1.21 |
| AT2G17440 | PIRL5 | plant intracellular ras group-related LRR 5 | **18.37** | 1.19 |
| AT5G18130 |  | Unknown | **18.33** |  |
| AT4G07820 |  | CAP (Cysteine-rich secretory proteins, Antigen 5, and Pathogenesis-related 1 protein) superfamily protein | **18.3** | 1.19 |
| AT3G06380 | TLP9 | tubby-like protein 9 | **18.29** | 0.91 |
| AT1G50430 | 7RED | Ergosterol biosynthesis ERG4/ERG24 family | **18.17** |  |
| AT5G14880 |  | Potassium transporter family protein | **18.12** |  |
| AT2G02960 |  | RING/FYVE/PHD zinc finger superfamily protein | **18.06** |  |
| AT4G22010 | sks4 | SKU5 similar 4 | **17.95** | 0.99 |
| AT1G32170 | XTH30 | xyloglucan endotransglucosylase/hydrolase 30 | **17.95** |  |
| AT5G63850 | AAP4 | amino acid permease 4 | **17.95** |  |
| AT1G62570 | FMO GS-OX4 | flavin-monooxygenase glucosinolate S-oxygenase 4 | **17.9** | 0.98 |
| AT1G80440 |  | Galactose oxidase/kelch repeat superfamily protein | **17.84** |  |
| AT4G39660 | AGT2 | alanine:glyoxylate aminotransferase 2 | **17.8** | 1.04 |
| AT3G54770 |  | RNA-binding (RRM/RBD/RNP motifs) family protein | **17.78** | 0.86 |
| AT1G08510 | FATB | fatty acyl-ACP thioesterases B | **17.25** | 1.16 |
| AT1G08920 | ESL1 | ERD (early response to dehydration) six-like 1 | **17.17** |  |
| AT4G10310 | HKT1 | high-affinity K+ transporter 1 | **17.11** |  |
| AT1G64370 |  | Unknown | **17.09** | 1.18 |
| AT3G03870 |  | Unknown | **17.01** |  |
| AT1G54030 | GOLD36 | GDSL-like Lipase/Acylhydrolase superfamily protein | **16.92** | 1.11 |
| AT3G23560 | ALF5 | MATE efflux family protein | **16.9** |  |
| AT3G19390 |  | Granulin repeat cysteine protease family protein | **16.88** | 1.09 |
| AT1G01750 | ADF11 | actin depolymerizing factor 11 | **16.87** | 0.88 |
| AT3G22370 | AOX1A | alternative oxidase 1A | **16.86** | 1.10 |
| AT4G36980 |  | Unknown | **16.76** |  |
| AT3G10190 |  | Calcium-binding EF-hand family protein | **16.61** | 1.10 |
| AT2G46140 |  | Late embryogenesis abundant protein | **16.59** | 0.85 |
| AT2G30440 | Plsp2B | thylakoid processing peptide | **16.58** | 1.03 |
| AT3G05020 | ACP | acyl carrier protein 1 | **16.55** | 0.96 |
| AT5G18520 |  | Lung seven transmembrane receptor family protein | **16.52** | 1.02 |
| AT1G12240 | BETAFRUCT4 | Glycosyl hydrolases family 32 protein | **16.46** | 1.16 |
| AT5G13330 | Rap2.6L | related to AP2 6l | **16.41** | 1.14 |
| AT2G15480 | UGT73B5 | UDP-glucosyl transferase 73B5 | **16.38** | 1.18 |
| AT3G03990 |  | alpha/beta-Hydrolases superfamily protein | **16.32** | 1.18 |
| AT5G48110 |  | Terpenoid cyclases/Protein prenyltransferases superfamily protein | **16.32** |  |
| AT1G62710 | BETA-VPE | beta vacuolar processing enzyme | **-16.29** | 0.86 |
| AT4G08685 | SAH7 | Pollen Ole e 1 allergen and extensin family protein | **-16.37** |  |
| AT3G24770 | CLE41 | CLAVATA3/ESR-RELATED 41 | **-16.39** |  |
| AT2G33590 |  | NAD(P)-binding Rossmann-fold superfamily protein | **-16.42** | 1.20 |
| AT4G09650 | PD | ATP synthase delta-subunit gene | **-16.51** | 0.90 |
| AT4G26130 |  | Unknown | **-16.53** | 0.97 |
| AT2G32990 | GH9B8 | glycosyl hydrolase 9B8 | **-16.53** |  |
| AT1G03130 | PSAD-2 | photosystem I subunit D-2 | **-16.58** | 0.75 |
| AT5G26742 | emb1138 | DEAD box RNA helicase (RH3) | **-16.59** | 1.03 |
| AT1G72060 |  | serine-type endopeptidase inhibitors | **-16.59** |  |
| AT3G14420 |  | Aldolase-type TIM barrel family protein | **-16.64** | 0.91 |
| AT2G33800 | EMB3113 | Ribosomal protein S5 family protein | **-16.64** | 0.94 |
| AT2G42220 |  | Rhodanese/Cell cycle control phosphatase superfamily protein | **-16.76** | 0.88 |
| AT3G62740 | BGLU7 | beta glucosidase 7 | **-16.78** |  |
| AT5G26670 |  | Pectinacetylesterase family protein | **-16.81** | 1.09 |
| AT2G44670 |  | Protein of unknown function (DUF581) | **-16.81** |  |
| AT4G11420 | EIF3A-1 | eukaryotic translation initiation factor 3A | **-16.85** | 1.05 |
| AT2G46820 | PSAP | photosystem I P subunit | **-16.9** | 1.02 |
| AT1G52420 |  | UDP-Glycosyltransferase superfamily protein | **-16.92** | 0.89 |
| AT1G45130 | BGAL5 | beta-galactosidase 5 | **-16.95** | 0.99 |
| AT1G75280 |  | NmrA-like negative transcriptional regulator family protein | **-16.99** | 0.80 |
| AT3G62030 | CYP20-3 | rotamase CYP 4 | **-17.1** | 0.94 |
| AT4G34160 | CYCD3 | CYCLIN D3;1 | **-17.13** |  |
| AT3G22320 | RPABC24.3 | Eukaryotic rpb5 RNA polymerase subunit family protein | **-17.28** | 1.02 |
| AT3G27570 |  | Sucrase/ferredoxin-like family protein | **-17.48** | 0.91 |
| AT5G19290 |  | alpha/beta-Hydrolases superfamily protein | **-17.56** | 0.99 |
| AT1G68010 | HPR1 | hydroxypyruvate reductase | **-17.7** | 1.03 |
| AT3G60520 |  | Unknown | **-17.73** |  |
| AT3G48670 | IDN2 | XH/XS domain-containing protein | **-17.76** | 1.07 |
| AT5G64940 | H13 | ABC2 homolog 13 | **-17.85** | 1.19 |
| AT3G26710 | CCB1 | cofactor assembly of complex C | **-17.92** |  |
| AT5G43330 | c-NAD-MDH2 | Lactate/malate dehydrogenase family protein | **-18.25** | 0.77 |
| AT3G20100 | CYP705A19 | cytochrome P450, family 705, subfamily A, polypeptide 19 | **-18.28** | 1.18 |
| AT2G36910 | ABCB1 | ATP binding cassette subfamily B1 | **-18.3** | 1.12 |
| AT2G38370 |  | Plant protein of unknown function (DUF827) | **-18.33** |  |
| AT1G16850 |  | Unknown | **-18.38** | 0.74 |
| AT2G38710 |  | AMMECR1 family | **-18.42** | 0.92 |
| AT4G27260 | GH3.5 | Auxin-responsive GH3 family protein | **-18.59** |  |
| AT3G01390 | AVMA10 | vacuolar membrane ATPase 10 | **-18.74** | 0.92 |
| AT3G46780 | PTAC16 | plastid transcriptionally active 16 | **-18.8** | 0.85 |
| AT4G18020 | APRR2 | CheY-like two-component responsive regulator family protein | **-18.86** |  |
| AT1G51630 |  | O-fucosyltransferase family protein | **-18.89** | 0.95 |
| AT5G01810 | PK10 | CBL-interacting protein kinase 15 | **-18.9** | 0.99 |
| AT1G49560 |  | Homeodomain-like superfamily protein | **-18.92** |  |
| AT3G23290 | LSH4 | Protein of unknown function (DUF640) | **-18.93** |  |
| AT2G40480 |  | Plant protein of unknown function (DUF827) | **-18.99** |  |
| AT3G13730 | CYP90D1 | cytochrome P450, family 90, subfamily D, polypeptide 1 | **-19.06** | 1.08 |
| AT1G07880 | MPK13 | Protein kinase superfamily protein | **-19.06** |  |
| AT2G43420 |  | 3-beta hydroxysteroid dehydrogenase/isomerase family protein | **-19.14** |  |
| AT1G22065 |  | Unknown | **-19.28** | 1.26 |
| AT2G23600 | ACL | acetone-cyanohydrin lyase | **-19.31** | 0.95 |
| AT4G35350 | XCP1 | xylem cysteine peptidase 1 | **-19.33** | 0.82 |
| AT5G54130 |  | Calcium-binding endonuclease/exonuclease/phosphatase family | **-19.56** |  |
| AT2G39870 |  | Unknown | **-19.58** | 0.93 |
| AT1G74520 | HVA22A | HVA22 homologue A | **-19.6** |  |
| AT2G33550 |  | Homeodomain-like superfamily protein | **-19.66** | 0.89 |
| AT5G63400 | ADK1 | adenylate kinase 1 | **-19.67** | 0.91 |
| AT5G58330 |  | lactate/malate dehydrogenase family protein | **-19.71** | 0.93 |
| AT4G04640 | PC1 | ATPase, F1 complex, gamma subunit protein | **-19.75** | 1.35 |
| AT5G12250 | TUB6 | beta-6 tubulin | **-19.78** | 0.94 |
| AT5G02160 |  | Unknown | **-19.88** | 0.87 |
| AT2G47560 |  | RING/U-box superfamily protein | **-19.88** |  |
| AT4G22780 | ACR7 | ACT domain repeat 7 | **-19.93** |  |
| AT1G19540 |  | NmrA-like negative transcriptional regulator family protein | **-20.04** | 0.91 |
| AT4G33950 | OST1 | Protein kinase superfamily protein | **-20.37** |  |
| AT5G61020 | ECT3 | evolutionarily conserved C-terminal region 3 | **-20.43** | 0.99 |
| AT4G37550 |  | Acetamidase/Formamidase family protein | **-20.52** | 1.14 |
| AT1G71980 |  | Protease-associated (PA) RING/U-box zinc finger family protein | **-20.63** | 0.97 |
| AT1G14720 | XTH28 | xyloglucan endotransglucosylase/hydrolase 28 | **-20.96** |  |
| AT1G52230 | PSAH-2 | photosystem I subunit H2 | **-20.98** | 0.72 |
| AT4G30650 |  | Low temperature and salt responsive protein family | **-21.04** |  |
| AT5G16250 |  | Unknown | **-21.05** |  |
| AT5G08530 | CI51 | 51 kDa subunit of complex I | **-21.11** | 0.99 |
| AT3G02770 |  | Ribonuclease E inhibitor RraA/Dimethylmenaquinone methyltransferase | **-21.2** | 0.85 |
| AT4G01050 | TROL | thylakoid rhodanese-like | **-21.24** | 0.89 |
| AT1G28130 | GH3.17 | Auxin-responsive GH3 family protein | **-21.27** | 1.19 |
| AT5G22090 |  | Protein of unknown function (DUF3049) | **-21.38** | 0.95 |
| AT3G22550 |  | Protein of unknown function (DUF581) | **-21.38** |  |
| AT2G14878 |  | Unknown | **-21.42** |  |
| AT3G26060 | PRX Q | Thioredoxin superfamily protein | **-21.48** | 0.90 |
| AT2G30590 | WRKY21 | WRKY DNA-binding protein 21 | **-21.48** |  |
| AT4G39730 |  | Lipase/lipooxygenase, PLAT/LH2 family protein | **-21.52** | 1.09 |
| AT3G19370 |  | Plant protein of unknown function (DUF869) | **-21.54** | 0.88 |
| AT3G51720 |  | Plant protein of unknown function (DUF827) | **-21.64** |  |
| AT4G26270 | PFK3 | phosphofructokinase 3 | **-21.65** | 0.98 |
| AT5G05790 |  | Duplicated homeodomain-like superfamily protein | **-21.81** |  |
| AT4G16980 |  | arabinogalactan-protein family | **-21.82** |  |
| AT2G39570 | ACR9 | ACT domain-containing protein | **-22.29** | 1.14 |
| AT3G61820 |  | Eukaryotic aspartyl protease family protein | **-22.34** | 0.75 |
| AT5G46730 |  | glycine-rich protein | **-22.41** |  |
| AT4G38932 |  | Unknown | **-22.43** |  |
| AT1G51400 |  | Photosystem II 5 kD protein | **-22.61** | 0.80 |
| AT5G61410 | EMB2728 | D-ribulose-5-phosphate-3-epimerase | **-22.8** | 0.95 |
| AT1G04680 |  | Pectin lyase-like superfamily protein | **-22.95** | 0.96 |
| AT4G01100 | ADNT1 | adenine nucleotide transporter 1 | **-23.12** | 1.03 |
| AT4G32690 | GLB3 | hemoglobin 3 | **-23.31** | 0.99 |
| AT3G07410 | RABA5b | RAB GTPase homolog A5B | **-23.41** |  |
| AT1G76160 | sks5 | SKU5 similar 5 | **-23.44** | 0.99 |
| AT2G14210 | AGL44 | AGAMOUS-like 44 | **-23.46** |  |
| AT3G25290 |  | Auxin-responsive family protein | **-23.48** | 0.83 |
| AT5G09620 |  | Octicosapeptide/Phox/Bem1p family protein | **-23.49** | 0.84 |
| AT4G24770 | RBP31 | 31-kDa RNA binding protein | **-23.59** | 0.92 |
| AT5G13630 | ABAR | magnesium-chelatase subunit chlH, chloroplast, putative / Mg-protoporphyrin IX chelatase, putative (CHLH) | **-23.67** | 1.05 |
| AT3G12780 | PGK1 | phosphoglycerate kinase 1 | **-23.93** | 0.93 |
| AT1G19330 |  | Unknown | **-23.98** |  |
| AT1G78450 |  | SOUL heme-binding family protein | **-23.99** | 0.95 |
| AT5G18840 |  | Major facilitator superfamily protein | **-24** |  |
| AT4G39720 |  | VQ motif-containing protein | **-24.22** |  |
| AT5G47110 | LIL3:2 | Chlorophyll A-B binding family protein | **-24.4** | 0.98 |
| AT1G67195 | MIR414 | MIR414 (MICRORNA 414) | **-24.45** |  |
| AT1G62790 |  | Bifunctional inhibitor/lipid-transfer protein/seed storage 2S albumin superfamily protein | **-24.59** | 1.02 |
| AT5G09810 | ACT7 | actin 7 | **-24.62** | 0.99 |
| AT2G33620 |  | AT hook motif DNA-binding family protein | **-24.97** | 0.83 |
| AT2G17845 |  | NAD(P)-binding Rossmann-fold superfamily protein | **-24.98** |  |
| AT2G22080 |  | Unknown | **-25.28** |  |
| AT3G55770 | WLIM2b | GATA type zinc finger transcription factor family protein | **-25.36** | 1.05 |
| AT5G04950 | NAS1 | nicotianamine synthase 1 | **-25.53** | 0.82 |
| AT3G27310 | PUX1 | plant UBX domain-containing protein 1 | **-25.53** | 0.95 |
| AT1G54660 |  | Unknown | **-26.06** |  |
| AT5G66190 | LFNR1 | ferredoxin-NADP(+)-oxidoreductase 1 | **-26.21** | 1.20 |
| AT3G06435 |  | Expressed protein | **-26.25** |  |
| AT2G44360 |  | Unknown | **-26.38** | 0.80 |
| AT1G15270 |  | Translation machinery associated TMA7 | **-26.63** | 0.96 |
| AT1G14290 | SBH2 | sphingoid base hydroxylase 2 | **-26.64** |  |
| AT1G07140 | SIRANBP | Pleckstrin homology (PH) domain superfamily protein | **-26.71** | 1.06 |
| AT4G31020 |  | alpha/beta-Hydrolases superfamily protein | **-26.87** |  |
| AT5G10580 |  | Protein of unknown function, DUF599 | **-26.91** |  |
| AT2G23610 | MES3 | methyl esterase 3 | **-26.92** | 0.91 |
| AT3G53460 | CP29 | chloroplast RNA-binding protein 29 | **-27** | 0.87 |
| AT2G21330 | FBA1 | fructose-bisphosphate aldolase 1 | **-27.05** | 0.81 |
| AT1G70370 | PG2 | polygalacturonase 2 | **-27.31** | 0.88 |
| AT1G59960 |  | NAD(P)-linked oxidoreductase superfamily protein | **-27.49** | 1.34 |
| AT5G13140 |  | Pollen Ole e 1 allergen and extensin family protein | **-27.53** |  |
| AT2G10410 | SADHU1-1 | transposable element gene | **-27.56** |  |
| AT2G20260 | PSAE-2 | photosystem I subunit E-2 | **-27.77** | 0.81 |
| AT3G03020 |  | Unknown | **-27.81** |  |
| AT2G46080 |  | Unknown | **-27.98** |  |
| AT1G60710 | B2 | NAD(P)-linked oxidoreductase superfamily protein | **-28.15** | 1.00 |
| AT2G19990 | PR-1-LIKE | pathogenesis-related protein-1-like | **-28.25** | 0.72 |
| AT3G03380 | DEG7 | DegP protease 7 | **-28.35** | 0.97 |
| AT5G61440 | ACHT5 | atypical CYS HIS rich thioredoxin 5 | **-28.44** |  |
| AT1G20270 |  | 2-oxoglutarate (2OG) and Fe(II)-dependent oxygenase superfamily protein | **-28.51** | 0.95 |
| AT2G33850 |  | Unknown | **-28.62** | 0.77 |
| AT3G48560 | AHAS | chlorsulfuron/imidazolinone resistant 1 | **-28.66** | 0.93 |
| AT5G42200 |  | RING/U-box superfamily protein | **-28.72** |  |
| AT1G32470 |  | Single hybrid motif superfamily protein | **-28.77** | 0.88 |
| AT1G70310 | SPDS2 | spermidine synthase 2 | **-28.93** | 0.97 |
| AT1G55810 | UKL3 | uridine kinase-like 3 | **-29.47** | 0.92 |
| AT4G37930 | SHM1 | serine transhydroxymethyltransferase 1 | **-29.56** | 0.93 |
| AT1G67740 | PSBY | photosystem II BY | **-29.75** |  |
| AT3G03780 | MS2 | methionine synthase 2 | **-30.03** | 1.32 |
| AT1G56580 | SVB | Protein of unknown function, DUF538 | **-30.23** | 0.87 |
| AT5G53880 |  | Unknown | **-30.41** |  |
| AT2G41530 | SFGH | S-formylglutathione hydrolase | **-30.46** | 1.00 |
| AT2G28780 |  | Unknown | **-30.55** |  |
| AT4G32260 | PDE334 | ATPase, F0 complex, subunit B/B', bacterial/chloroplast | **-30.64** | 0.91 |
| AT1G44575 | CP22 | Chlorophyll A-B binding family protein | **-30.84** | 1.04 |
| AT4G29310 |  | Protein of unknown function (DUF1005) | **-31.02** |  |
| AT1G68238 |  | Unknown | **-31.1** |  |
| AT1G47960 | C/VIF1 | cell wall / vacuolar inhibitor of fructosidase 1 | **-31.2** | 0.80 |
| AT4G18700 | WL4 | CBL-interacting protein kinase 12 | **-31.27** | 0.90 |
| AT5G40780 | LHT1 | lysine histidine transporter 1 | **-31.6** | 0.97 |
| AT5G26710 |  | Glutamyl/glutaminyl-tRNA synthetase, class Ic | **-31.82** | 1.27 |
| AT4G21620 |  | glycine-rich protein | **-32.03** |  |
| AT4G15780 | VAMP724 | vesicle-associated membrane protein 724 | **-32.23** |  |
| AT4G24026 |  | Unknown | **-32.84** |  |
| AT3G16140 | PSAH-1 | photosystem I subunit H-1 | **-32.9** | 0.87 |
| AT1G43790 | TED6 | tracheary element differentiation-related 6 | **-33.04** |  |
| AT1G22220 | AUF2 | F-box family protein | **-33.08** | 0.86 |
| AT5G62740 | HIR1 | SPFH/Band 7/PHB domain-containing membrane-associated protein family | **-33.58** | 1.02 |
| AT1G42970 | GAPB | glyceraldehyde-3-phosphate dehydrogenase B subunit | **-33.63** | 1.04 |
| AT2G32240 |  | Unknown | **-33.99** | 0.96 |
| AT4G36430 |  | Peroxidase superfamily protein | **-34** | 0.83 |
| AT1G77150 |  | Pentatricopeptide repeat (PPR) superfamily protein | **-34.34** |  |
| AT2G19800 | MIOX2 | myo-inositol oxygenase 2 | **-34.38** |  |
| AT1G32060 | PRK | phosphoribulokinase | **-34.94** | 0.95 |
| AT3G47720 | SRO4 | similar to RCD one 4 | **-34.96** |  |
| AT1G55210 |  | Disease resistance-responsive (dirigent-like protein) family protein | **-35.11** | 0.88 |
| AT5G51550 | EXL3 | EXORDIUM like 3 | **-35.12** | 0.90 |
| AT3G23170 |  | Unknown | **-35.38** |  |
| AT5G59320 | LTP3 | lipid transfer protein 3 | **-35.39** | 0.74 |
| AT5G62390 | BAG7 | BCL-2-associated athanogene 7 | **-35.93** | 1.19 |
| AT2G05070 | LHCB2 | photosystem II light harvesting complex gene 2.2 | **-36.05** |  |
| AT5G54770 | THI1 | thiazole biosynthetic enzyme, chloroplast (ARA6) (THI1) (THI4) | **-36.14** | 0.81 |
| AT3G28480 |  | Oxoglutarate/iron-dependent oxygenase | **-36.25** | 0.91 |
| AT1G43710 | emb1075 | Pyridoxal phosphate (PLP)-dependent transferases superfamily protein | **-36.26** | 0.93 |
| AT4G35790 | PLDDELTA | phospholipase D delta | **-36.3** | 1.17 |
| AT2G15960 |  | Unknown | **-36.97** | 1.11 |
| AT3G13650 |  | Disease resistance-responsive (dirigent-like protein) family protein | **-37.45** | 0.77 |
| AT1G12900 | GAPA-2 | glyceraldehyde 3-phosphate dehydrogenase A subunit 2 | **-37.94** | 1.01 |
| AT4G17670 |  | Protein of unknown function (DUF581) | **-38.08** |  |
| AT1G72430 |  | SAUR-like auxin-responsive protein family | **-38.26** |  |
| AT5G37720 | ALY4 | ALWAYS EARLY 4 | **-38.65** | 1.16 |
| AT2G21170 | PDTPI | triosephosphate isomerase | **-38.87** | 0.95 |
| AT3G28200 |  | Peroxidase superfamily protein | **-38.88** | 0.89 |
| AT5G50200 | NRT3.1 | nitrate transmembrane transporters | **-39.08** | 0.85 |
| AT4G03280 | PETC | photosynthetic electron transfer C | **-39.14** | 0.74 |
| AT1G12810 |  | proline-rich family protein | **-39.18** |  |
| AT4G34950 |  | Major facilitator superfamily protein | **-39.23** |  |
| AT2G36570 |  | Leucine-rich repeat protein kinase family protein | **-39.45** | 0.92 |
| AT2G42610 | LSH10 | Protein of unknown function (DUF640) | **-39.48** | 0.83 |
| AT2G05100 | LHCB2 | photosystem II light harvesting complex gene 2.1 | **-39.79** | 0.87 |
| AT1G03600 | PSB27 | photosystem II family protein | **-39.8** | 0.79 |
| AT4G01150 |  | Unknown | **-40.19** | 0.91 |
| AT3G55610 | P5CS2 | delta 1-pyrroline-5-carboxylate synthase 2 | **-40.26** | 1.09 |
| AT5G49720 | GH9A1 | glycosyl hydrolase 9A1 | **-40.57** | 0.98 |
| AT5G12140 | CYS1 | cystatin-1 | **-40.63** | 0.87 |
| AT2G18150 |  | Peroxidase superfamily protein | **-40.64** | 0.85 |
| AT2G31360 | ADS2 | 16:0delta9 desaturase 2 | **-42.25** |  |
| AT4G05150 |  | Octicosapeptide/Phox/Bem1p family protein | **-42.28** | 0.88 |
| AT3G20390 |  | endoribonuclease L-PSP family protein | **-42.64** | 0.89 |
| AT4G21280 | PSBQ | photosystem II subunit QA | **-42.95** | 0.79 |
| AT3G09820 | ADK1 | adenosine kinase 1 | **-43** | 0.90 |
| AT5G43150 |  | Unknown | **-43.56** |  |
| AT3G23000 | SR2 | CBL-interacting protein kinase 7 | **-43.68** | 0.80 |
| AT4G17260 |  | Lactate/malate dehydrogenase family protein | **-43.97** | 0.78 |
| AT1G60950 | FD2 | 2Fe-2S ferredoxin-like superfamily protein | **-44.5** | 0.91 |
| AT3G21055 | PSBTN | photosystem II subunit T | **-45.86** | 0.74 |
| AT3G08030 |  | Protein of unknown function, DUF642 | **-46.16** | 0.80 |
| AT1G74940 |  | Protein of unknown function (DUF581) | **-46.66** |  |
| AT4G34620 | SSR16 | small subunit ribosomal protein 16 | **-47.79** | 0.93 |
| AT1G15820 | CP24 | light harvesting complex photosystem II subunit 6 | **-48.07** | 1.03 |
| AT4G30140 | CDEF1 | GDSL-like Lipase/Acylhydrolase superfamily protein | **-48.31** | 0.89 |
| AT1G69530 | #¡REF! | expansin A1 | **-48.44** | 1.03 |
| AT4G37800 | XTH7 | xyloglucan endotransglucosylase/hydrolase 7 | **-48.62** | 0.97 |
| AT4G03210 | XTH9 | xyloglucan endotransglucosylase/hydrolase 9 | **-48.71** | 0.84 |
| AT4G38970 | FBA2 | fructose-bisphosphate aldolase 2 | **-49.19** | 0.89 |
| AT2G21870 | MGP1 | copper ion binding;cobalt ion binding;zinc ion binding | **-49.3** | 1.20 |
| AT2G26500 |  | cytochrome b6f complex subunit (petM), putative | **-49.41** |  |
| AT1G26270 |  | Phosphatidylinositol 3- and 4-kinase family protein | **-50.26** |  |
| AT3G59910 |  | Ankyrin repeat family protein | **-50.75** |  |
| AT3G19130 | RBP47B | RNA-binding protein 47B | **-50.83** | 0.77 |
| AT5G46110 | APE2 | Glucose-6-phosphate/phosphate translocator-related | **-50.94** | 1.01 |
| AT2G19970 |  | CAP (Cysteine-rich secretory proteins, Antigen 5, and Pathogenesis-related 1 protein) superfamily protein | **-51.93** | 0.79 |
| AT3G60750 |  | Transketolase | **-52.41** | 1.28 |
| AT3G08940 | LHCB4.2 | light harvesting complex photosystem II | **-52.46** | 0.96 |
| AT5G03545 | IPS2 | Unknown | **-53.28** |  |
| AT3G54890 | LHCA1 | photosystem I light harvesting complex gene 1 | **-53.58** | 0.91 |
| AT2G44080 | ARL | ARGOS-like | **-55.48** |  |
| AT5G22740 | CSLA02 | cellulose synthase-like A02 | **-55.99** | 1.04 |
| AT1G61520 | LHCA3 | photosystem I light harvesting complex gene 3 | **-56.35** | 0.81 |
| AT5G07030 |  | Eukaryotic aspartyl protease family protein | **-57.29** | 0.76 |
| AT2G47520 | ERF71 | Integrase-type DNA-binding superfamily protein | **-57.98** |  |
| AT1G20340 | DRT112 | Cupredoxin superfamily protein | **-58.42** | 0.81 |
| AT2G01140 | PDE345 | Aldolase superfamily protein | **-59.01** | 0.91 |
| AT5G38410 |  | Ribulose bisphosphate carboxylase (small chain) family protein | **-59.24** |  |
| AT1G12780 | UGE1 | UDP-D-glucose/UDP-D-galactose 4-epimerase 1 | **-60.2** | 0.86 |
| AT3G57520 | SIP2 | seed imbibition 2 | **-60.64** | 0.92 |
| AT4G31730 | GDU1 | glutamine dumper 1 | **-60.96** |  |
| AT2G24762 | GDU4 | glutamine dumper 4 | **-61.32** |  |
| AT3G56940 | ACSF | dicarboxylate diiron protein, putative (Crd1) | **-61.56** | 1.33 |
| AT4G12800 | PSAL | photosystem I subunit l | **-61.76** | 0.84 |
| AT4G13235 | EDA21 | embryo sac development arrest 21 | **-61.79** | 0.93 |
| AT1G74470 |  | Pyridine nucleotide-disulphide oxidoreductase family protein | **-62.55** | 1.07 |
| AT2G30570 | PSBW | photosystem II reaction center W | **-62.72** |  |
| AT2G47710 |  | Adenine nucleotide alpha hydrolases-like superfamily protein | **-64** | 0.77 |
| AT3G29970 |  | B12D protein | **-64.9** |  |
| AT1G31330 | PSAF | photosystem I subunit F | **-67.05** | 0.85 |
| AT1G08380 | PSAO | photosystem I subunit O | **-67.2** | 0.95 |
| AT5G65140 | TPPJ | Haloacid dehalogenase-like hydrolase (HAD) superfamily protein | **-70.93** |  |
| AT5G24770 | VSP2 | vegetative storage protein 2 | **-74.7** | 0.71 |
| AT5G47060 |  | Protein of unknown function (DUF581) | **-74.98** |  |
| AT5G54270 | LHCB3 | light-harvesting chlorophyll B-binding protein 3 | **-75.34** | 0.95 |
| AT3G30775 | POX | Methylenetetrahydrofolate reductase family protein | **-76.31** | 0.88 |
| AT2G06520 | PSBX | photosystem II subunit X | **-77.31** |  |
| AT5G01530 | LHCB4.1 | light harvesting complex photosystem II | **-78.17** | 1.04 |
| AT1G72360 | ERF73 | Integrase-type DNA-binding superfamily protein | **-79.12** |  |
| AT1G04410 | c-NAD-MDH1 | Lactate/malate dehydrogenase family protein | **-80.5** | 0.84 |
| AT1G55670 | PSAG | photosystem I subunit G | **-83.33** | 0.99 |
| AT3G61470 | LHCA2 | photosystem I light harvesting complex gene 2 | **-84.36** | 0.89 |
| AT5G44730 |  | Haloacid dehalogenase-like hydrolase (HAD) superfamily protein | **-85.88** | 0.88 |
| AT5G65640 | bHLH093 | beta HLH protein 93 | **-87.88** | 0.92 |
| AT2G39730 | RCA | rubisco activase | **-87.93** | 0.88 |
| AT1G19600 |  | pfkB-like carbohydrate kinase family protein | **-88.71** | 0.84 |
| AT2G36530 | ENO2 | Enolase | **-89.97** | 0.89 |
| AT1G33055 |  | Unknown | **-90.39** |  |
| AT2G39510 |  | nodulin MtN21 /EamA-like transporter family protein | **-91.65** |  |
| AT1G06680 | OE23 | photosystem II subunit P-1 | **-91.79** | 0.76 |
| AT1G11680 | CYP51 | CYTOCHROME P450 51G1 | **-92.82** | 1.09 |
| AT4G02970 | 7SL-1 | 7SL RNA1 | **-93.85** |  |
| AT4G17940 |  | Tetratricopeptide repeat (TPR)-like superfamily protein | **-93.93** |  |
| AT2G36580 |  | Pyruvate kinase family protein | **-95.38** | 1.22 |
| AT5G54960 | PDC2 | pyruvate decarboxylase-2 | **-95.53** | 1.06 |
| AT4G30190 | AHA2 | H(+)-ATPase 2 | **-98.49** | 1.07 |
| AT3G26650 | GAPA | glyceraldehyde 3-phosphate dehydrogenase A subunit | **-98.66** | 1.31 |
| AT2G34390 | NIP2;1 | NOD26-like intrinsic protein 2;1 | **-105.06** |  |
| AT2G39770 | CYT1 | Glucose-1-phosphate adenylyltransferase family protein | **-110.9** | 1.00 |
| AT3G18280 |  | Bifunctional inhibitor/lipid-transfer protein/seed storage 2S albumin superfamily protein | **-110.97** | 0.78 |
| AT3G47470 | CAB4 | light-harvesting chlorophyll-protein complex I subunit A4 | **-113** | 0.93 |
| AT5G10050 |  | NAD(P)-binding Rossmann-fold superfamily protein | **-114.92** | 0.99 |
| AT2G41430 | CID1 | dehydration-induced protein (ERD15) | **-119.02** | 0.83 |
| AT4G28250 | EXPB3 | expansin B3 | **-119.03** | 0.96 |
| AT1G28400 |  | Unknown | **-119.83** | 0.80 |
| AT1G30510 | RFNR2 | root FNR 2 | **-123.53** | 0.91 |
| AT3G02885 | GASA5 | GAST1 protein homolog 5 | **-124.56** | 1.02 |
| AT5G39890 |  | Protein of unknown function (DUF1637) | **-131.71** |  |
| AT5G66570 | MSP-1 | PS II oxygen-evolving complex 1 | **-133.64** | 0.77 |
| AT4G10340 | LHCB5 | light harvesting complex of photosystem II 5 | **-136.41** | 0.90 |
| AT3G49120 | PCB | peroxidase CB | **-143.69** | 1.01 |
| AT5G48485 | DIR1 | Bifunctional inhibitor/lipid-transfer protein/seed storage 2S albumin superfamily protein | **-145.26** | 0.84 |
| AT4G02290 | GH9B13 | glycosyl hydrolase 9B13 | **-146.27** | 0.95 |
| AT1G77760 | GNR1 | nitrate reductase 1 | **-149.28** | 1.00 |
| AT3G27220 |  | Galactose oxidase/kelch repeat superfamily protein | **-149.81** |  |
| AT1G17290 | AlaAT1 | alanine aminotransferas | **-151.09** | 0.89 |
| AT4G24110 |  | Unknown | **-152.47** |  |
| AT1G12805 |  | nucleotide binding | **-152.75** |  |
| AT2G34420 | LHB1B2 | photosystem II light harvesting complex gene B1B2 | **-154.71** | 0.73 |
| AT4G10270 |  | Wound-responsive family protein | **-154.84** |  |
| AT4G25100 | FSD1 | Fe superoxide dismutase 1 | **-158.32** | 0.97 |
| AT2G39700 | EXP4 | expansin A4 | **-168.2** | 0.75 |
| AT3G45160 |  | Putative membrane lipoprotein | **-168.5** |  |
| AT5G05960 |  | Bifunctional inhibitor/lipid-transfer protein/seed storage 2S albumin superfamily protein | **-168.75** | 0.93 |
| AT3G22120 | CWLP | cell wall-plasma membrane linker protein | **-177.4** | 0.99 |
| AT2G16600 | ROC3 | rotamase CYP 3 | **-178.31** | 0.80 |
| AT2G14247 | IRP3 | Expressed protein | **-183.86** |  |
| AT5G66985 |  | Unknown | **-187.13** | 0.81 |
| AT5G19140 | AILP1 | Aluminium induced protein with YGL and LRDR motifs | **-200.94** | 0.84 |
| AT1G64720 | CP5 | Polyketide cyclase/dehydrase and lipid transport superfamily protein | **-210.31** |  |
| AT1G35720 | ANNAT1 | annexin 1 | **-211.98** | 1.35 |
| AT1G11840 | GLX1 | glyoxalase I homolog | **-213.81** | 1.36 |
| AT1G19570 | DHAR1 | dehydroascorbate reductase | **-214.01** | 0.81 |
| AT3G54260 | TBL36 | TRICHOME BIREFRINGENCE-LIKE 36 | **-217.29** |  |
| AT3G02550 | LBD41 | LOB domain-containing protein 41 | **-221.5** | 1.14 |
| AT1G08090 | ACH1 | nitrate transporter 2:1 | **-223.6** | 0.81 |
| AT2G30490 | C4H | cinnamate-4-hydroxylase | **-223.97** | 1.20 |
| AT1G76930 | EXT1 | extensin 4 | **-237.65** |  |
| AT2G02130 | LCR68 | low-molecular-weight cysteine-rich 68 | **-241.89** | 0.91 |
| AT3G27770 |  | Unknown | **-243.98** |  |
| AT4G21960 | PRXR1 | Peroxidase superfamily protein | **-254.98** | 0.85 |
| AT2G19590 | ACO1 | ACC oxidase 1 | **-280.44** | 0.88 |
| AT5G10040 |  | Unknown | **-297.7** |  |
| AT1G37130 | NR2 | nitrate reductase 2 | **-323.63** | 1.15 |
| AT2G38380 |  | Peroxidase superfamily protein | **-324.92** | 0.89 |
| AT2G31390 |  | pfkB-like carbohydrate kinase family protein | **-332.9** | 0.75 |
| AT1G67090 | RBCS1A | ribulose bisphosphate carboxylase small chain 1A | **-341.12** | 0.85 |
| AT3G41768 |  | rRNA | **-379.83** |  |
| AT4G33560 |  | Wound-responsive family protein | **-403.46** | 0.76 |
| AT5G20830 | ASUS1 | sucrose synthase 1 | **-510.91** | 0.95 |
| AT1G11580 | PMEPCRA | methylesterase PCR A | **-514.45** | 0.85 |
| AT3G43190 | SUS4 | sucrose synthase 4 | **-673.27** | 0.87 |
| AT1G43800 |  | Plant stearoyl-acyl-carrier-protein desaturase family protein | **-745.95** | 0.77 |
| AT1G29930 | AB140 | chlorophyll A/B binding protein 1 | **-755.74** |  |
| AT2G01021 |  | Unknown | **-850.77** |  |
| AT5G15230 | GASA4 | GAST1 protein homolog 4 | **-857.11** | 1.12 |
| AT1G13440 | GAPC-2 | glyceraldehyde-3-phosphate dehydrogenase C2 | **-1286.09** | 0.94 |
